# Singlet Fission in Pyrene‐Fused Azaacene Dimers

**DOI:** 10.1002/anie.201911529

**Published:** 2019-11-27

**Authors:** Juan P. Mora‐Fuentes, Ilias Papadopoulos, Dominik Thiel, Roberto Álvarez‐Boto, Diego Cortizo‐Lacalle, Timothy Clark, Manuel Melle‐Franco, Dirk M. Guldi, Aurelio Mateo‐Alonso

**Affiliations:** ^1^ POLYMAT University of the Basque Country UPV/EHU Avenida de Tolosa 72 20018 Donostia-San Sebastian Spain; ^2^ Department of Chemistry and Pharmacy & Interdisciplinary Center for Molecular Materials Friedrich-Alexander-Universität Erlangen-Nürnberg Egerlandstr. 3 91058 Erlangen Germany; ^3^ CICECO—Aveiro Institute of Materials Department of Chemistry University of Aveiro 3810-193 Aveiro Portugal; ^4^ Computer-Chemistry Centre Department of Chemistry and Pharmacy Friedrich-Alexander-Universität Erlangen-Nürnberg Naegelsbachstr. 25 91052 Erlangen Germany; ^5^ Ikerbasque Basque Foundation for Science Bilbao Spain

**Keywords:** nitrogen doping, polycyclic aromatic hydrocarbons, pyrene-fused azaacenes, singlet fission, tetracene

## Abstract

Singlet fission has emerged as a promising strategy to avoid the loss of extra energy through thermalization in solar cells. A family of dimers consisting of nitrogen‐doped pyrene‐fused acenes that undergo singlet fission with triplet quantum yields as high as 125 % are presented. They provide new perspectives for nitrogenated polycyclic aromatic hydrocarbons and for the design of new materials for singlet fission.

## Graphical abstract

**Come Together**: By introducing dibenzodiazahexacene moieties in a dimeric architecture, a new type of singlet fission material was obtained. Solvent‐dependent triplet quantum yields of up to 125 % were achieved.

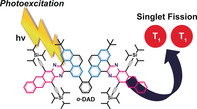

Singlet fission (SF) has emerged as a promising strategy to overcome the Shockley–Queisser limit that predicts a maximum power conversion efficiency of 33.7 % for a single junction device.^[1]^ This is because, in SF, a singlet excited state (S_1_) splits into two triplet excited states (T_1_) and hence the generation of the extra exciton per absorbed photon avoids the loss of extra energy through thermalization. SF has been observed in several families of conjugated molecules and polycyclic aromatic hydrocarbons,^[2]^ among which the acene family have shown a prominent position. For instance, acene dimers have become key to understanding how structural and electronic factors influence the dynamics of the formation and decay of triplet excitons.^[3]^ Since SF efficiencies are known to increase with the number of linearly fused rings and pentacene is the most stable of the higher acenes, the synthesis and study of pentacene dimers have received a lot of attention,[^2a^, ^2b^] among which maximum triplet quantum yields (TQYs) of 200 % have been reported.

However, the triplet energy of pentacene is not well‐matched for use in solar cells,^[4]^ and also, the stability of pentacene derivatives is limited in comparison to the shorter acenes.^[5]^ On this regard, tetracene has been investigated as an alternative since it shows triplet energies above the band gap of silicon[^4a^, ^4b^] and is known to be more stable than pentacene.^[5]^ Furthermore, dimers constituted by tetracene have been recently reported with TQYs ≥100 %.^[6]^

Substitutional nitrogen‐doping is another potential approach to improving the properties of acenes for SF, since the resulting azaacenes are more stable than the corresponding acenes and also their energy levels can be tuned with the number and the position of the nitrogen atoms.^[7]^ Nevertheless, azaacenes remain practically unexplored in SF and only a few studies about monomeric azaacenes in thin films have been reported.^[8]^

Herein, we report a family of dimers constituted by nitrogen‐doped pyrene‐fused acenes that undergo SF (Figure 1). This series of regioisomeric dimers are constituted by two dibenzodiazahexacene cores bridged by a phenylene linker in an *ortho*‐, *meta*‐, or *para*‐ substitution pattern (*o*‐DAD, *m*‐DAD, and *p*‐DAD), which show the electronic structure of two diazatetracenes (highlighted in pink) as the result of the localization of sextets in the off‐linear pyrene rings (highlighted in sky blue) that compartmentalize the acene backbone in smaller and more stable tetracenic residues. The combination of ultrafast transient absorption measurements with global and target analyses reveal TQYs as high as 125 % and a detailed SF mechanistic insight for pyrene‐fused azaacenes, which altogether provide new perspectives not only for azaacenes and nitrogenated pyrene‐fused acenes, but also for the design of new materials with enhanced properties for SF.

**Figure 1:**
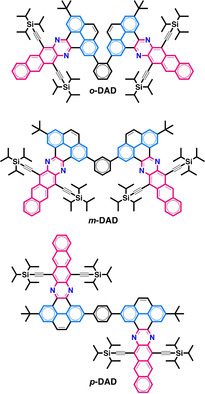
Chemical structures of *o*‐DAD, *m*‐DAD, and *p*‐DAD.

Dibenzodiazahexacene dimers *o*‐DAD, *m*‐DAD, and *p*‐DAD are members of the pyrene‐fused azaacene^[9]^ family that combine linear annulations (acene backbone) with periodic off‐linear bisannulations (pyrene residues). Pyrene‐fused azaacenes have shown a high stability as illustrated by synthesis of monodisperse nanoribbons with up to 30 linearly fused rings^[10]^ and of polydisperse nanoribbons with approximately 80 linearly fused rings.^[11]^ We selected dibenzodiazahexacene derivatives with TIPS‐acetylene substituents because of their tetracene electronic structure, their stability, and solubility.^[12]^ *o*‐DAD, *m*‐DAD, and *p*‐DAD were synthesized following the route set out in Scheme 1. A key intermediate is the synthesis of pyrene dione **A** that will enable the synthesis of the dibenzodiazahexacene core and also the coupling with other units. This structure is quite challenging as it is a completely asymmetric pyrene derivative. To obtain this intermediate, we developed a new synthetic route with pyrene as the starting point that combines desymmetrization across the 2‐ and 7‐positions and across the K‐regions. The first step is the introduction of a *tert*‐butyl group in position 2 of pyrene by Friedel–Crafts alkylation.^[13]^ Then, 2‐*tert*‐butylpyrene **B** was borylated on the 7‐position through an iridium‐catalyzed reaction (63 %). Subsequently, we carried out the oxidation of one of the K‐regions of pyrene **C** using NaIO_4_ and RuCl_3_ as a catalyst, which to our surprise worked very well (53 %), highlighting the broad functional group compatibility of this reaction. Also the cyclocondensation of dione **A** and anthracenediamine **D** ^[7c]^ was satisfactory (56 %), yielding the asymmetric dibenzodiazahexacene **E** with a boronate ester that can be further engaged in cross‐coupling reactions. Suzuki cross coupling reactions between **E** and diiodobenzene with conventional heating led to poor yields <7 %. Conversely, when the reactions were carried out with microwave heating higher yields were obtained in shorter reaction times. The *o*‐DAD, *m*‐DAD, and *p*‐DAD dimers were obtained via microwave‐assisted Suzuki cross‐coupling reaction between the precursor **E** with *ortho*‐, *meta*, and *para*‐diiodobenzene affording, respectively the desired *ortho*‐, *meta*, and *para* compounds (25 %, 45 %, and 23 % for *o*‐DAD, *m*‐DAD, and *p*‐DAD, respectively). For reference purposes dibenzoazahexacene (DAM) monomer was prepared by condensation of 2,7‐di‐*tert*‐butyl pyrene‐4,5‐dione^[13]^ and anthracenediamine **D**.^[7c]^ Complete synthetic procedures including ^1^H NMR, ^13^C NMR, matrix‐assisted laser desorption/ionization time‐of‐flight high‐resolution mass spectrometry (MALDI‐TOF HRMS) and a X‐ray crystal structure for DAM are provided in the Supporting Information.

**Scheme 1:**
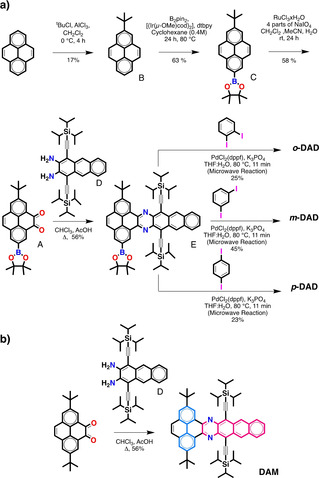
a) Synthesis of dimers (*o*‐DAD, *m*‐DAD, and *p*‐DAD). b) Synthesis of reference monomer (DAM).

To shed light onto electronic interactions between the two dibenzoazahexacenes in the different dimers, we first turned to steady‐state investigations in solvents with different polarity, namely toluene, THF, and benzonitrile (Figure 2 a; Supporting Information, Figure S1 and Table S1). Similar absorption spectra were recorded for DAM, *o*‐DAD, *m*‐DAD, and *p*‐DAD with a series of maxima in the 300–425 and the 450–700 nm ranges (Figure 2 a). A closer look at the 588 and 640 nm maxima reveals that, on one hand, *o*‐DAD, *m*‐DAD, and *p*‐DAD are red‐shifted in comparison to DAM. The 478, 506, and 540 nm maxima, on the other hand, are blue‐shifted in *m*‐DAD and *p*‐DAD and red‐shifted on *o*‐DAD. The extinction coefficients of *o*‐DAD, *m*‐DAD, and *p*‐DAD are not doubled in comparison to that of the reference DAM. This speaks for electronic interactions in the ground state as seen for pentacene dimers.^[14]^ The computed TD‐DFT spectra at B3LYP‐6–311+g(2d,p)/PBEh‐3c level (Supporting Information, Figure S2) and at the M06‐2X‐toluene‐6–311+g(2d,p)/PBEh‐3c level (Supporting Information, Figure S3) show the same trends as the experimental spectra. In both cases, the first absorption peak position is essentially the same for all dimers, and appears at slightly lower energies than DAM; meanwhile, the second transition appears red‐shifted for *o*‐DAD, in agreement with the experimental spectra.

**Figure 2:**
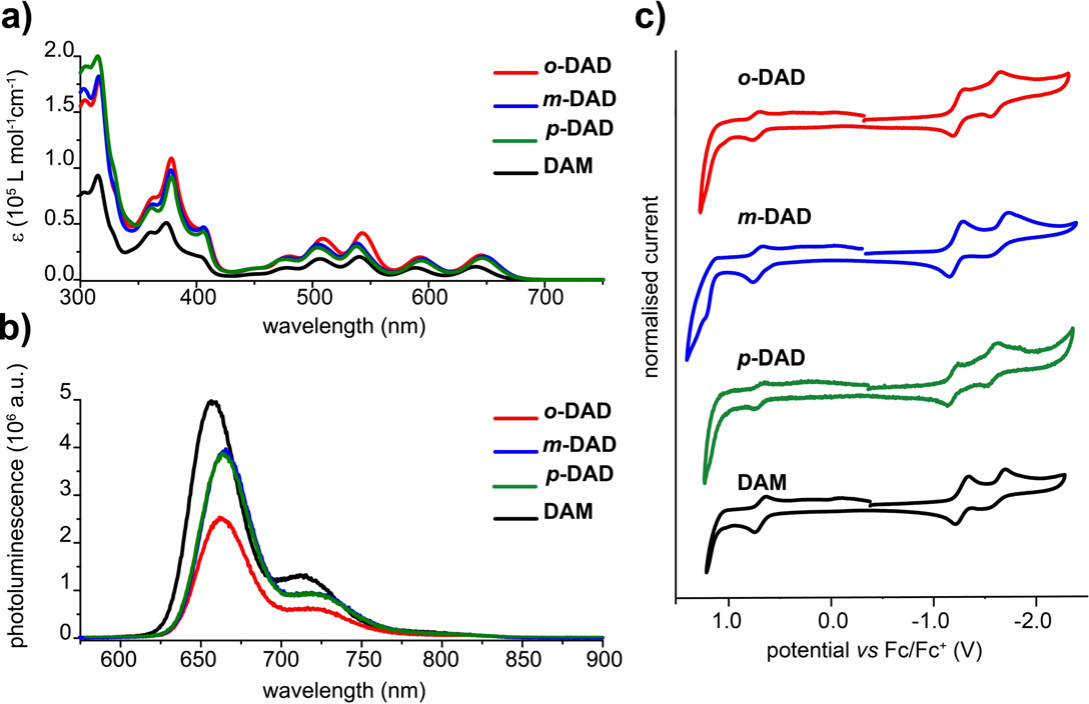
a) Absorption and b) fluorescence (photoexcitation at 550 nm with an optical density of 0.025) spectra of DAM, *o*‐DAD, *m*‐DAD, and *p*‐DAD in toluene. c) Cyclic voltammograms of DAM, *o*‐DAD, *m*‐DAD, and *p*‐DAD in an Ar‐saturated 0.1 m solution of *n*Bu_4_NPF_6_ in CH_2_Cl_2_.

In the electronic excited state (Figure 2 b; Supporting Information, Figure S4 and Table S1), the interactions are even more evident and more dependent on the solvent polarity. DAM fluoresces in the red to NIR range with quantum yields of 25 %, 22 %, and 16 % in toluene, THF, and benzonitrile, respectively. The vibrational fine structure as seen in toluene (Figure 2 b) gradually disappears when inspecting THF and benzonitrile (Supporting Information, Figure S4). *o*‐DAD, *m*‐DAD, and *p*‐DAD all fluoresce, but with much lower quantum yields than DAM: 13 %, 20 %, and 19 %, respectively, in toluene (Figure 2 b). At this point, we relate the fluorescence quenching to the interactions between the two dibenzoazahexacenes, which intensifies from *o*‐DAD to *p*‐DAD and *m*‐DAD. In time correlated single photon counting (TCSPC) measurements with DAM only a single, moderately long‐lived component of 26.0 ns was found in toluene. Interestingly, *o*‐DAD and *p*‐DAD revealed three components rather than one, that is, contributions from a short‐, an intermediate‐, and a long‐lived species.^[15]^ What was 26.0 ns in DAM, is 1.7 and 15.9 ns for *o*‐DAD and *p*‐DAD as the short‐lived species, and 21.8 ns for *m*‐DAD as the intermediate‐lived species, respectively, in toluene. In benzonitrile, the lifetimes were as short as 1.0 ns in, for example, *o*‐DAD (Supporting Information, Figures S5–S8 and Table S2).^[16]^

The electrochemical properties of DAM, *o*‐DAD, *m*‐DAD, and *p*‐DAD were studied by cyclic voltammetry in an Ar‐saturated solution of CH_2_Cl_2_ with *n*Bu_4_NPF_6_ as electrolyte (Figure 2 c). The oxidation and reduction potentials are given versus the ferrocene/ferrocenium (Fc/Fc^+^) couple that was used as an internal standard. The voltammograms of all dimers and the reference monomer show two reduction waves and one oxidation wave with no remarkable differences in the half‐wave potentials (Supporting Information, Table S3).

The energy levels were estimated from the electronic absorption and electrochemical measurements (Supporting Information, Tables S1 and S3), which correlate well with the DFT calculations (Supporting Information, Table S4). The optical HOMO–LUMO gaps (*E*_gap_^opt^≈1.8 eV) and electrochemical HOMO–LUMO gaps (*E*_gap_^CV^≈1.8 eV) are nearly identical for DAM, *o*‐DAD, *m*‐DAD, and *p*‐DAD. The electrochemical LUMO levels (or electron affinities) and the electrochemical HOMO levels (or ionization potentials), which were estimated from the onset of the first reduction and the oxidation potential, respectively, are also nearly identical for DAM, *o*‐DAD, *m*‐DAD, and *p*‐DAD (*E*_LUMO_≈−3.6 eV and *E*_HOMO_≈−5.4 eV). Electronic structure calculations yielded very similar relative results with subtle differences for the four studied molecules (Supporting Information, Table S4). The computed 2T_1_ is similar to S_1_ for B3LYP in vacuum and remarkably similar for the M06‐2X Hamiltonian computed in toluene (Supporting Information, Table S5) for all dimers, which therefore fulfil the energetic requirements for SF.

Femtosecond (fs‐TAS) and nanosecond transient absorption (ns‐TAS) measurements were performed to gather a more detailed understanding of the excited‐state dynamics. Fitting the differential absorption data with a combination of multiwavelength and target analyses sheds light onto lifetimes, quantum yields, and species associated spectra (SAS). In the case of DAM (Supporting Information, Figures S9–S12), 505 nm excitation experiments are best fit by a kinetic model that is based on the sequential formation of three species, namely (S_1_), (S_1_)_SOL_, and (T_1_) (Supporting Information, Figure S13 and Table S6). TQYs of 12 % and 8 % for DAM in toluene and benzonitrile were, respectively obtained.^[17]^ To establish the triplet excited state features, we turned to triplet–triplet energy‐transfer experiments using *N*‐methylfulleropyrrolidine (*N*‐MFP, 8.0×10^−5^ m) as a triplet sensitizer using 480 nm (800 nJ) photoexcitation (Supporting Information, Figures S14–S17).

Fitting the fs‐TAS and ns‐TAS data of *m*‐DAD (Supporting Information, Figures S18–S21) required the use of a four‐species kinetic model (Supporting Information, Figure S22 and Table S7). After excitation at 505 nm, the singlet excited state (S_1_S_0_) undergoes solvent reorganization to afford (S_1_S_0_)_SOL_ that transforms quickly and directly into a correlated triplet excited state ^1^(T_1_T_1_). The last step in the sequence is spin decoherence that results in the uncorrelated triplet excited state (T_1_+T_1_) (Supporting Information, Figure S22 and Table S7). TQYs for ^1^(T_1_T_1_) and (T_1_+T_1_) are 70 % and 27 % in toluene and 30 % and 32 % in benzonitrile, respectively.

Fitting the fs‐TAS/ ns‐TAS data for *o*‐DAD (Figure 3 a,b; Supporting Information, Figures S23–S26 and Table S8) and *p*‐DAD (Supporting Information, Figures S27–S30 and Table S9) necessitated the modifications of the deactivation mechanism. In this case, (S_1_S_0_)_CT_, which is a virtual intermediate in *m*‐DAD, becomes a real intermediate in *o*‐DAD and *p*‐DAD. In turn, a five species kinetic model is applied (Figure 3 c). The deconvoluted fs‐ and ns‐TAS spectra derived from target analysis are illustrated as a leading example for *o*‐DAD (Figure 3 a,b; Supporting Information, Figures S23–S26). 505 nm excitation leads to the instantaneous formation of (S_1_S_0_) followed by solvent reorganization. The correspondingly formed (S_1_S_0_)_SOL_ transitions in a parallel fashion to ^1^(T_1_T_1_); a fast and direct pathway, on one hand, as well as a slow and indirect pathway. Implicit in the indirect pathway is (S_1_S_0_)_CT_ as a real intermediate on the way towards ^1^(T_1_T_1_); this is the extra species relative to the kinetic model used for *m*‐DAD.[^14a^, ^18^] ^1^(T_1_T_1_) TQYs in toluene of 125 % and 82 % for *o*‐DAD and *p*‐DAD, respectively, corroborate SF in the deactivation of them. Increasing the polarity leads along with shorter lifetimes to lower TQYs of 92 % for *o*‐DAD and 35 % for *p*‐DAD. The immediate outcome of using benzonitrile is an energetic lowering of the real intermediate (S_1_S_0_)_CT_. As a matter of fact, we note that this pathway intensifies from 50 % in toluene to 70 % in benzonitrile. Furthermore, the equilibrium between (S_1_S_0_)_CT_ and ^1^(T_1_T_1_) is heavily shifted towards the former rather than the latter and, in turn, the direct deactivation is favored. TQYs of (T_1_+T_1_) remain in all cases at around 30 %.

**Figure 3:**
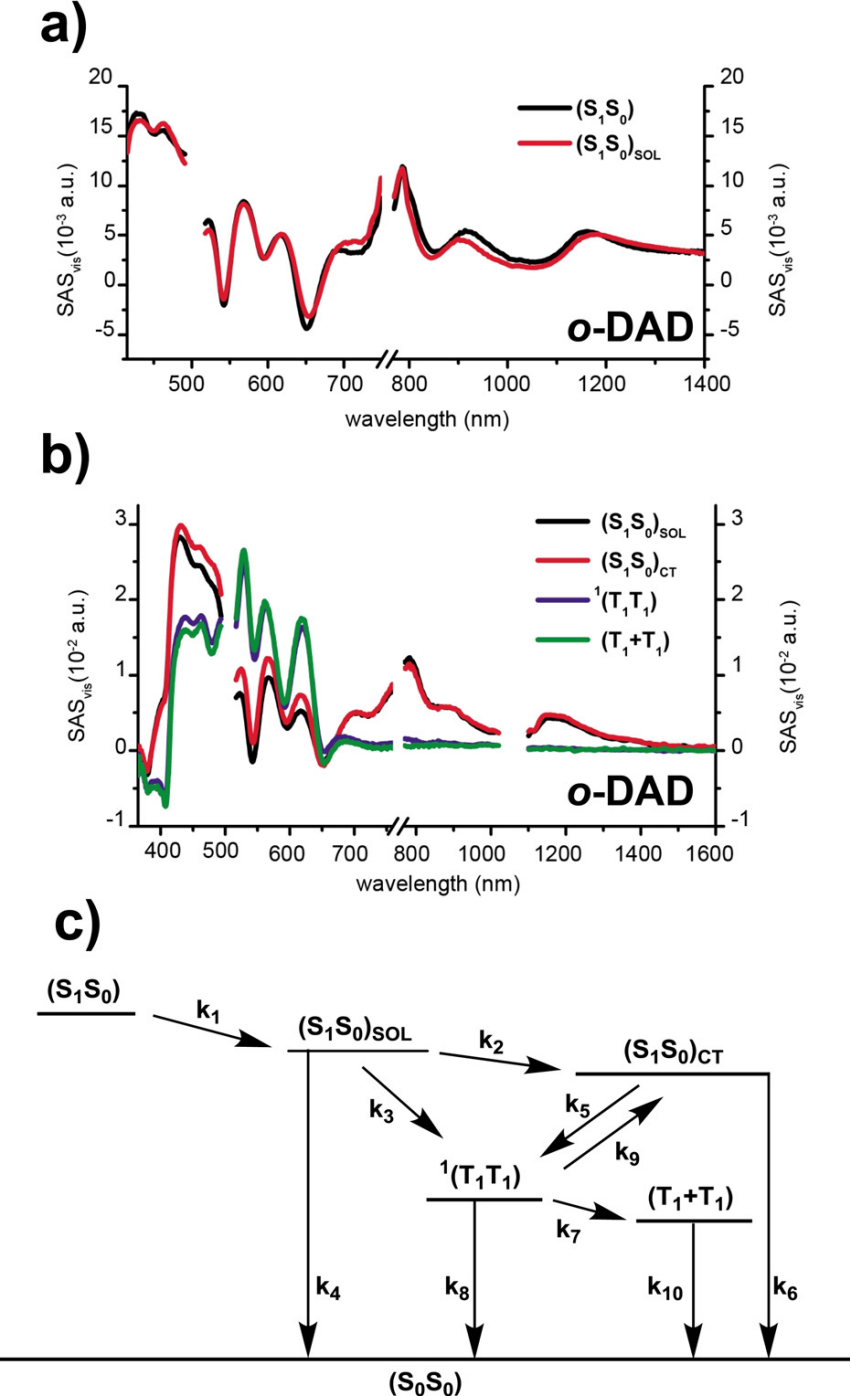
a) Deconvoluted fs‐TA spectra of the singlet excited (S_1_S_0_) (black), and the stabilized singlet excited state (S_1_S_0_)_SOL_ (red) of *o*‐DAD as obtained by target analysis in toluene. b) Deconvoluted ns‐TA spectra of the stabilized singlet excited state (S_1_S_0_)_SOL_ (black), the intermediate CT‐state (S_1_S_0_)_CT_ (red), the singlet correlated triplet state ^1^(T_1_T_1_) (blue), and the uncorrelated triplet excited state (T_1_+T_1_) (green) of *o*‐DAD as obtained by target analysis in toluene. c) Kinetic model used to fit the transient absorption data for *o*‐DAD and *p*‐DAD.

A likely rationale for higher ^1^(T_1_T_1_) TQYs in *o*‐DAD and *p*‐DAD is the directing nature of *ortho*‐ and *para*‐substitution and, as such, the better electronic communication between tetracenes. Stronger coupling must be also responsible for the fact that the intermediate (S_1_S_0_)_CT_ state is only observable in *o*‐DAD and *p*‐DAD, while it is a virtual state in *m*‐DAD. Finally, the close spatial proximity between the two dibenzoazahexacenes in *o*‐DAD results in an increase of electronic communication and enables the highest TQYs. The latter is supported by the experimental and calculated (M06‐2X‐toluene‐6–311+g(2d,p)/PBEh‐3c) electronic absorption spectra (Figure 2 a; Supporting Information, Figures S2 and S3) that illustrate how the direct contact between tetracene units in *o*‐DAD has a strong effect on the S_0_→S_2_ transition, which combines contributions with a sizable charge‐transfer (CT) character owing to the transfer of charge between the two different aromatic tetracene moieties (Supporting Information, Figures S31–S33). While in the case of *m*‐DAD and *p*‐DAD the S_0_→S_2_ transition is not red‐shifted because the two tetracene units are spatially decoupled (Figure 2 a; Supporting Information, Figures S31 and S32). This direct contact is also reflected experimentally by the higher fluorescence quenching on *o*‐DAD (Figure 2 b). To check the DFT findings, we also computed the excited states of the *o*‐DAD‐H model system (this is an *o*‐DAD model in which the TIPS groups have been exchanged by H) at the NEVPT2(8,8)/def‐SV(P) level. This yielded two local excitation (LE) states at 2.28 and 2.39 eV and two darker CT states at 2.36 and 2.37 eV, which confirms the M06‐2X finding yet also suggesting that these CT states might be nearly degenerate to the LE S_1_ states. Also, at this theory level, the T_1_ state appears at 1.23 eV, which is also roughly at half the S_1_ energy.

In conclusion, we have reported the synthesis of a new family of regioisomeric diazatetracene dimers constituted of two dibenzodiazahexacene units linked by a phenylene ring in an *ortho*, *meta*, and *para* substitution pattern. In *o*‐DAD the two tetracenes are in close contact, while in the case of *m*‐DAD and *p*‐DAD the two tetracenes are spatially decoupled, as demonstrated by a combination of electronic absorption and photoluminescence measurements and theoretical calculations. Femtosecond (fs‐TAS) and nanosecond transient absorption (ns‐TAS) measurements reveal how the enhanced electronic communication in *o*‐DAD gives rise to a TQY >100 %. Overall this work provides new application perspectives for pyrene‐fused azaacenes, but also for the design of new materials with enhanced properties for SF, in which the triplet energy and the stability are better suited for use in solar cells.

## Conflict of interest

The authors declare no conflict of interest.

## Supporting information

As a service to our authors and readers, this journal provides supporting information supplied by the authors. Such materials are peer reviewed and may be re‐organized for online delivery, but are not copy‐edited or typeset. Technical support issues arising from supporting information (other than missing files) should be addressed to the authors.

Supplementary
