# Supplementary material for: Singlet Fission in Pyrene‐Fused Azaacene Dimers
Source: Angew Chem Int Ed Engl. 2019 Nov 27;59(3):1113–7. doi: 10.1002/anie.201911529 (PMC7687256; doi:10.1002/anie.201911529)
Supplement: Supplementary file 1 — Supplementary [file ANIE-59-1113-s001.pdf]

Angewandte Chemie
Eine Zeitschrift der Gesellschaft Deutscher Chemiker

Supporting Information

Singlet Fission in Pyrene-Fused Azaacene Dimers

Juan P. Mora-Fuentes[+], Ilias Papadopoulos[+], Dominik Thiel, Roberto Álvarez-Boto, Diego Cortizo-Lacalle, Timothy Clark, Manuel Melle-Franco, Dirk M. Guldi,* and Aurelio Mateo-Alonso**

anie_201911529_sm_miscellaneous_information.pdf

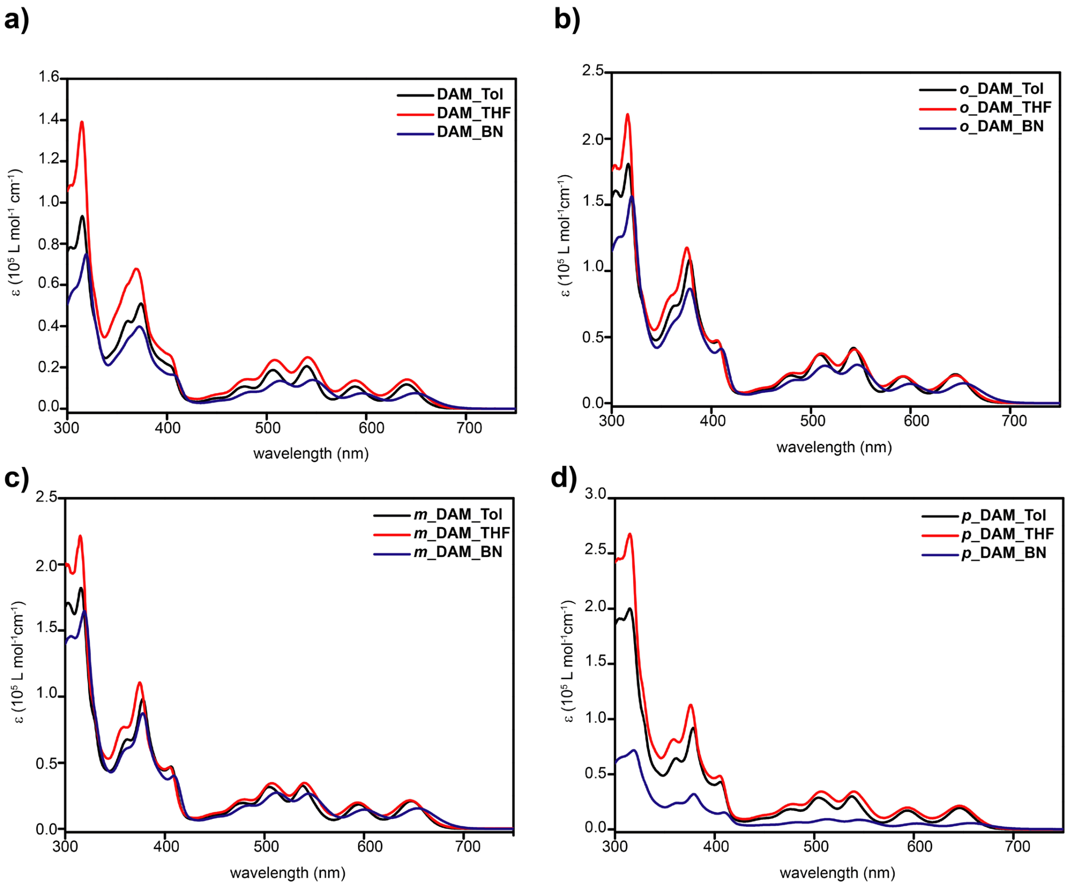

Figure S1. Absorption electronic spectra of a) **DAM**, b) ***o*-DAD**, c) ***m*-DAD**, d) ***p*-DAD** in toluene (Tol), THF and benzonitrile (BN).

Table S1. Optoelectronic properties of **DAM**, ***o*-DAD**, ***m*-DAD**, ***p*-DAD** in toluene (Tol), THF and benzonitrile (BN).

	Abs (nm)	λ_{onset} (nm)	E_{gap}^{opt} (eV)	ε M^{-1} cm^{-1}	Em (nm)	QY (%)
**DAM_Tol**	640	668	1.85	11715	659	25
**DAM_THF**	641	673	1.84	14140	665	22
**DAM_BN**	649	690	1.79	7500	681	16
***o*-DAD_Tol**	645	670	1.85	21940	662	13
***o*-DAD_THF**	645.5	677	1.83	21040	670	11.5
***o*-DAD_Bn**	653	690	1.79	15085	686	7.5
***m*-DAD_Tol**	647.5	671	1.85	21000	665	20
***m*-DAD_THF**	646	677	1.83	21600	670	18
***m*-DAD_Bn**	654	690	1.79	15560	686	11
***p*-DAD_Tol**	646	672	1.84	19435	662	19
***p*-DAD_THF**	646	678	1.83	21375	670	15
***p*-DAD_THF**	657.5	704	1.76	5700	686	10

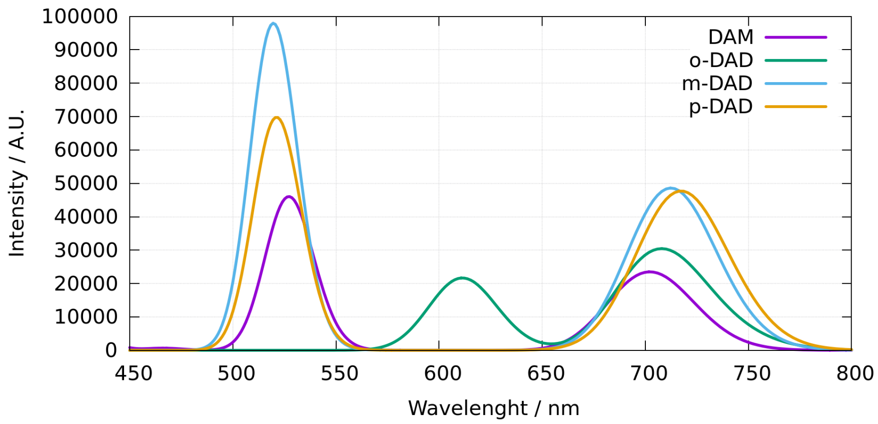

Figure S2. TD-DFT at the B3LYP-6-311+g(2d,p)/PBEh-3c level for **DAM**, ***o*-DAD**, ***m*-DAD** and ***p*-DAD** with 18 excitations.

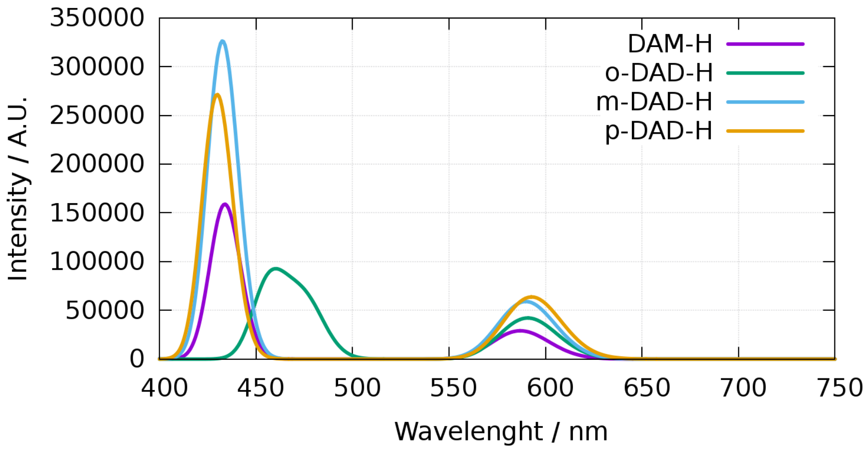

Figure S3. TD-DFT at the M06-2X-toluene-6-311+g(2d,p)/PBEh-3c level for **DAM-H**, ***o*-DAD-H**, ***m*-DAD-H** and ***p*-DAD-H** (this is a model system in which the TIPS and tert-butyl groups have been exchanged by H) molecules with 12 excitations.

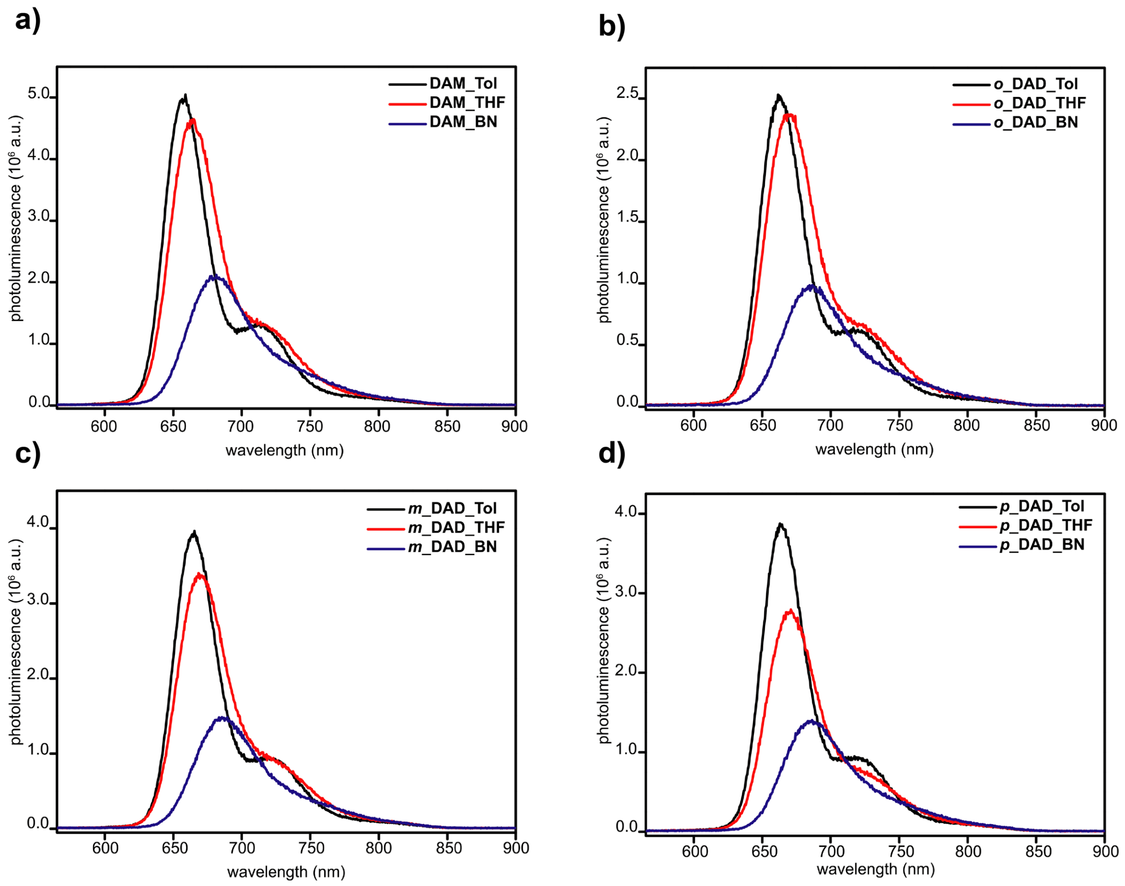

Figure S4. Photoluminiecence electronic spectra of a) **DAM**, b) ***o*-DAD**, c) ***m*-DAD**, d) ***p*-DAD** in toluene (Tol), THF and benzonitrile (BN).

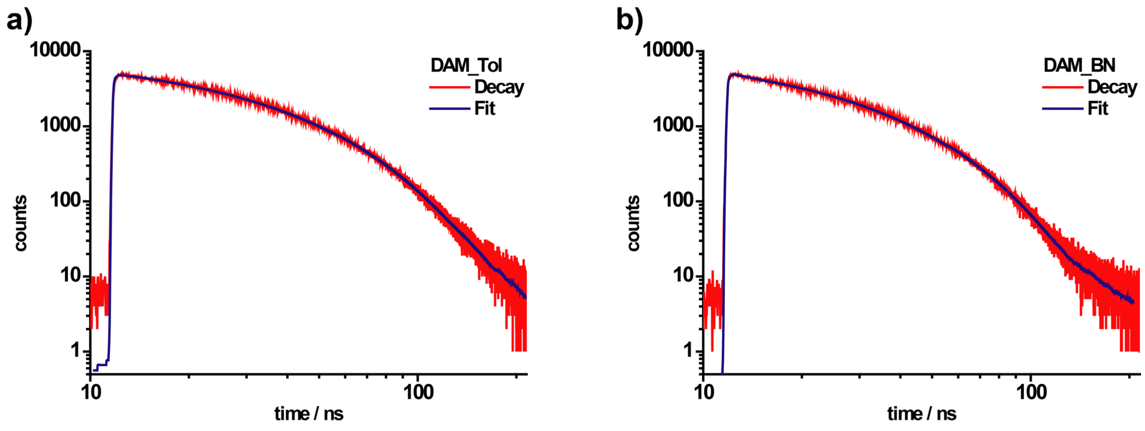

Figure S5. Fluorescence time profiles (red: decay data points; blue: fit) at an excitation of 505 nm of **DAM** in argon saturated a) toluene (Tol) and b) benzonitrile (BN) at room temperature.

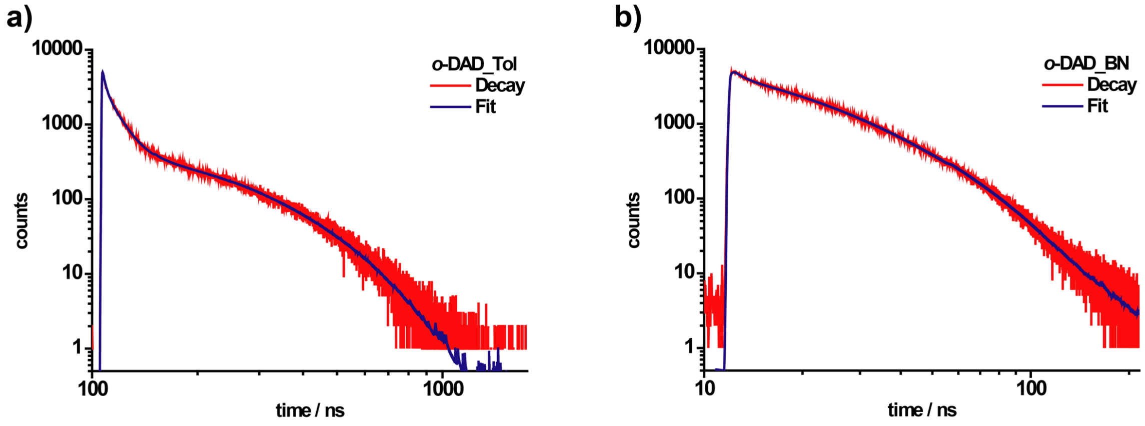

Figure S6. Fluorescence time profiles (red: decay data points; blue: fit) at an excitation of 505 nm of ***o*-DAD** in argon saturated a) toluene (Tol) and b) benzonitrile (BN) at room temperature.

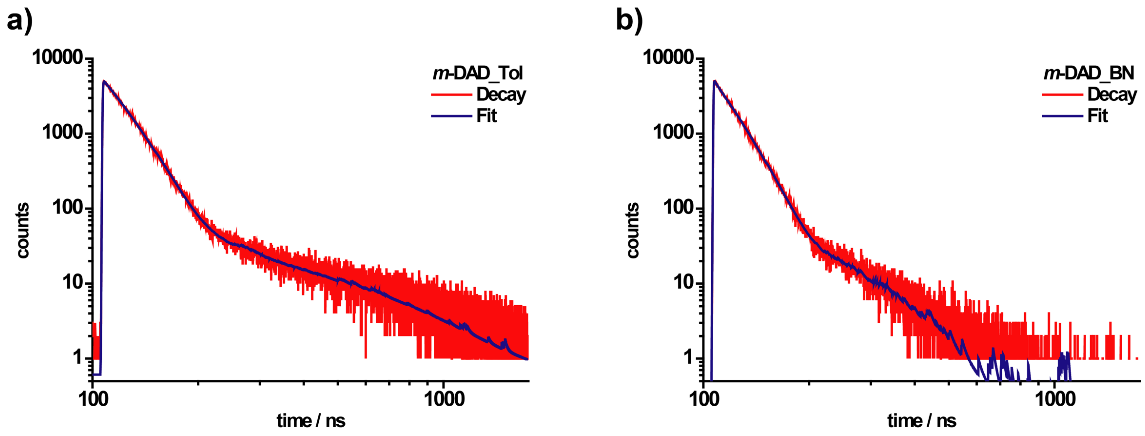

Figure S7. Fluorescence time profiles (red: decay data points; blue: fit) at an excitation of 505 nm of ***m*-DAD** in argon saturated a) toluene (Tol) and b) benzonitrile (BN) at room temperature.

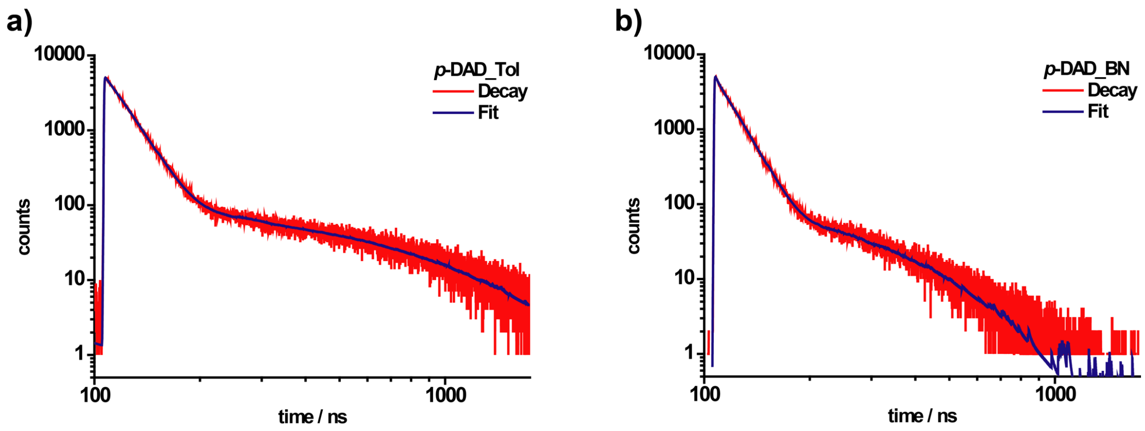

Figure S8. Fluorescence time profiles (red: decay data points; blue: fit) at an excitation of 505 nm of ***p*-DAD** in argon saturated a) toluene (Tol) and b) benzonitrile (BN) at room temperature.

Table S2. Fluorescence lifetimes, relative amplitudes, and chi-square values of **DAM**, ***o*-DAD**, ***m*-DAD**, and ***p*-DAD** in toluene (Tol) and benzonitrile (BN).

		τ_1 (ns) / Rel. A (%)	τ_2 (ns) / Rel. A (%)	τ_3 (ns) / Rel. A (%)	χ^2
**DAM**	**Tol**	26.01<br>100	-	-	1.3
	**BN**	20.24<br>100	-	-	1.2
***o*-DAD**	**Tol**	1.71<br>6.06	11.09<br>32.34	136.43<br>61.61	1.0
	**BN**	1.02<br>1.83	10.17<br>41.5	24.75<br>56.67	1.2
***m*-DAD**	**Tol**	21.83<br>86.75	471.95<br>13.25	-	1.1
	**BN**	16.78<br>93.56	108.91<br>6.44	-	1.0
***p*-DAD**	**Tol**	15.92<br>55.07	27.61<br>14.9	513.22<br>30.03	1.1
	**BN**	2.94<br>2.61	15.52<br>81.02	179.89<br>16.36	1.0

Table S3. Electrochemical properties of **DAM**, ***o*-DAD**, ***m*-DAD**, ***p*-DAD**.

	$E_{1/2}^{ox1}$ (V)	$E_{1/2}^{red1}$ (V)	$E_{1/2}^{red2}$ (V)	E_{gap}^{CV} (ev)	HOMO (eV)	LUMO (eV)
**DAM**	+0.69	–1.27	–1.60	1.77	–5.39	–3.62
***o*-DAD**	+0.73	–1.24	–1.59	1.78	–5.43	–3.65
***m*-DAD**	+0.71	–1.21	–1.63	1.74	–5.40	–3.66
***p*-DAD**	+0.69	–1.18	–1.55	1.74	–5.42	–3.68

Measured in 0.1 M $n\mathrm{Bu_4PF_6/CH_2Cl_2}$ (V), Ag wire pseudoreference electrode referenced *vs* SCE using ferrocene (Fc) as internal standard ($E_{1/2}^{Fc(SCE)}$ = +0.48 V). [d] Estimated from absorption onset (eV). [e] Estimated from CV E_{ONSET} (eV) according to E_{LUMO} = –4.8 –e(E_{ONSET} –$E_{1/2}^{Fc}$) where $E_{1/2}^{Fc}$ was measured in situ.

Table S4. Frontier orbitals computed with the B3LYP Hamiltonian with the 6-311+g(2d,p) basis set in vacuum for **DAM**, ***o*-DAD**, ***m*-DAD** and ***p*-DAD** and in toluene for **DAM-H**, ***o*-DAD-H**, ***m*-DAD-H** and ***p*-DAD-H** (this is a model system in which the TIPS and tert-butyl groups have been exchanged by H). **DAM**, ***o*-DAD**, ***m*-DAD** and ***p*-DAD** were optimized at PBEh-3c level while the hydrogens of **DAM-H**, ***o*-DAD-H**, ***m*-DAD-H** and ***p*-DAD-H** were optimized the B3LYP-6-31g(d,p) level. The colour code is: LUMOs, HOMOs and gaps. All values in eV.

B3LYP-vacuum-6-311+g(2d,p)/PBEh-3c	LUMO+2	LUMO+1	LUMO	HOMO	HOMO-1	HOMO-2	gap
**DAM**	-1.54	-1.73	**-3.14**	-5.23	-5.9	-6.32	2.09
***o*-DAD**	-1.84	-3.12	**-3.16**	-5.2	-5.23	-5.67	2.04
***m*-DAD**	-1.83	-3.19	**-3.19**	-5.26	-5.26	-5.99	2.07
***p*-DAD**	-1.82	-3.17	**-3.21**	-5.24	-5.28	-5.96	2.03
B3LYP-toluene-6-311+g(2d,p)/PBEh-3c							
**DAM-H**	-1.62	-1.78	-3.24	-5.36	-5.96	-6.56	2.1
***o*-DAD-H**	-1.83	-3.16	-3.19	-5.28	-5.29	-5.69	2.09
***m*-DAD-H**	-1.8	-3.24	-3.25	-5.35	-5.36	-5.98	2.1
***p*-DAD-H**	-1.8	-3.23	-3.25	-5.34	-5.36	-5.98	2.09

Table S5. Excitation energies to S1 and T1 states energy difference between S1 and 2T1 B3LYP Hamiltonian with the 6-311+g(2d,p) basis set in vacuum for **DAM**, ***o*-DAD**, ***m*-DAD** and ***p*-DAD** and in toluene for **DAM-H**, ***o*-DAD-H**, ***m*-DAD-H** and ***p*-DAM-H** (this is a model system in which the TIPS and tert-butyl groups have been exchanged by H). ***o*-DAD**, ***m*-DAD** and ***p*-DAD** were optimized at pbeH-3c level while the hydrogens of **DAM-H**, ***o*-DAD-H**, ***m*-DAD-H** and ***p*-DAM-H** geometries were optimized the B3LYP-6-31g(d,p) level. All values in eV.

B3LYP-6-311+g(2d,p)/PBEh-3c	T1	T1'	S1	S1'	S1-2T1
**DAM**	0.80		1.77		0.17
***o*-DAD**	0.77	0.79	1.73	1.76	0.19
***m*-DAD**	0.78	0.78	1.74	1.75	0.18
***p*-DAD**	0.78	0.79	1.71	1.74	0.16
M06-2X-Toluene-6-311g+g(2d,p)/PBEh-3c	T1	T1'	S1	S1'	S1-2T1
**DAM-H**	1.06		2.11		-0.01
***o*-DAD-H**	1.03	1.04	2.09	2.10	0.02
***m*-DAD-H**	1.05	1.06	2.10	2.11	-0.01
***p*-DAD-H**	1.05	1.05	2.09	2.11	0.00

It is within 10 ps that (S_1) transforms into the second species and this transformation is accompanied by hypsochromic as well as bathochromic shifts; most evidently when inspecting the 917 and 1180 nm features (Figure S9-S12). Solvent reorganization is the modus operandi for the (S_1)$_{SOL}$ formation as the dipole moment of (S_1) differs from that of (S_0). This is in sound agreement with recent work and reducing the solvation energy is the main driving force behind it.[1] From here on, intersystem crossing (ISC) is the mechanism, by which (S_1)$_{SOL}$ is converted into the third species, that is, the triplet excited (T_1) state. (T_1) features maxima at 437, 463, 529, 561, and 618 nm as well as minima at 380, 409, and 652 nm. From a comparison of the (S_1), (S_1)$_{SOL}$, and (T_1) characteristics, a significant overlap is noted both in terms of maxima and minima. Such an overlap hampers the TQY determination by using the intensification of the ground state bleaching.[1b, 2] The only notable exception is the 380 nm minimum, which was used to determine TQYs of 12% and 8% for **DAM** in toluene and benzonitrile, respectively.[3]

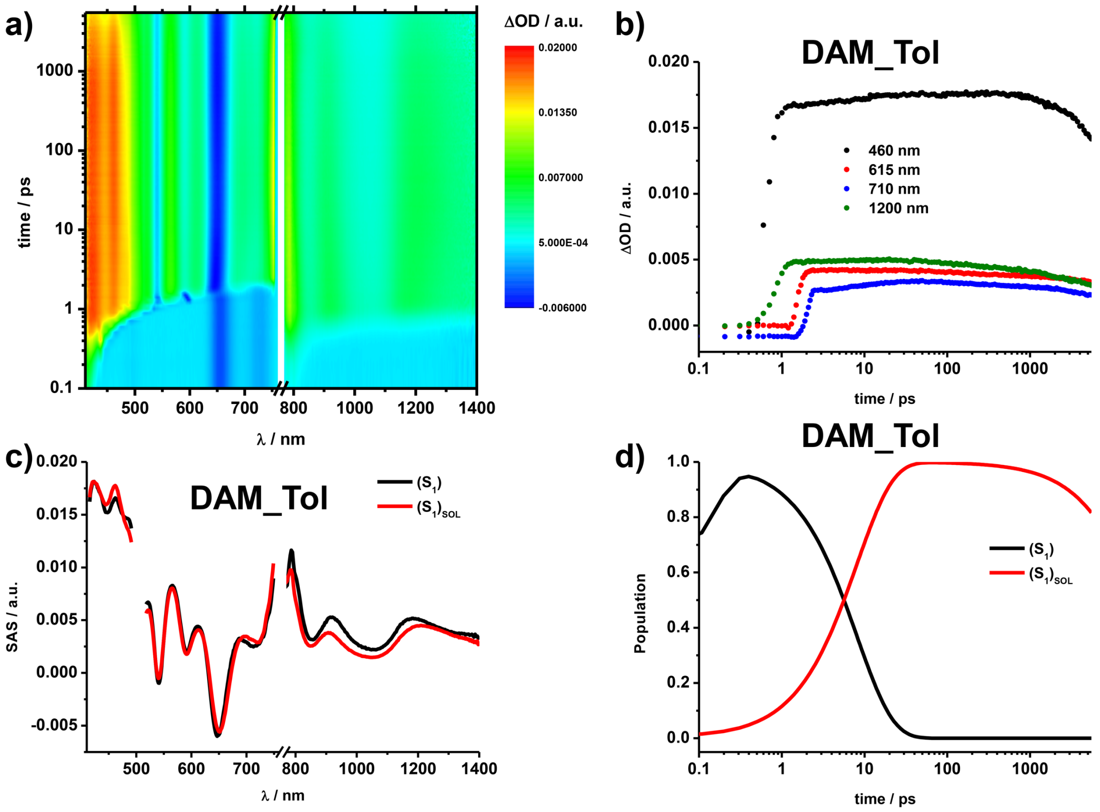

Figure S9. a) Differential fs-TA spectra (λ_{ex} = 505 nm; 500 nJ) of **DAM** in toluene (Tol) with time delays between 0 and 5 500 ps. b) Respective time absorption profiles at the given wavelengths. c) Deconvoluted fs-TA spectra of the singlet excited (S_1) (black) and the stabilized singlet excited state (S_1)$_{SOL}$ (red) of **DAM** as obtained by target analysis in toluene (Tol). d) Respective population kinetics. Please note that the (T_1) state was omitted in this figure, as it just begins to populate on this timescale and therefore cannot be completely deconvoluted.

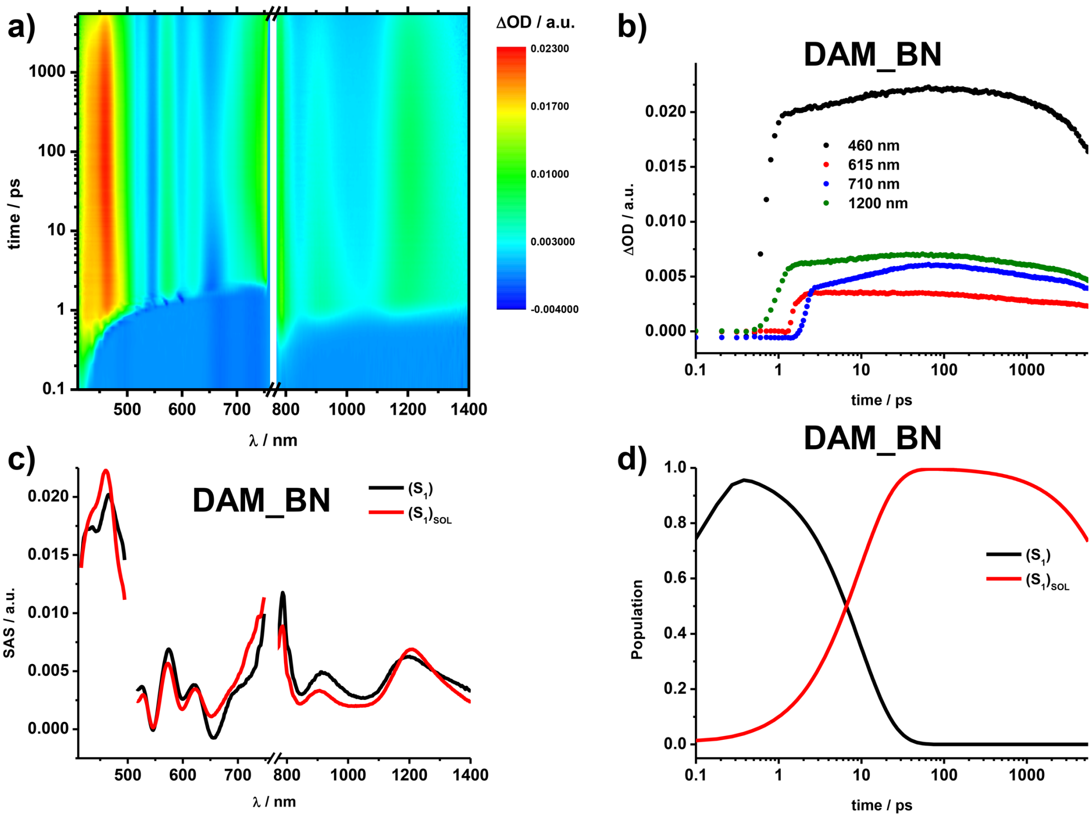

Figure S10. a) Differential fs-TA spectra (λ_{ex} = 505 nm; 500 nJ) of **DAM** in benzonitrile (BN) with time delays between 0 and 5 500 ps. b) Respective time absorption profiles at the given wavelengths. c) Deconvoluted fs-TA spectra of the singlet excited (S_1) (black) and the stabilized singlet excited state $(S_1)_{SOL}$ (red) of **DAM** as obtained by target analysis in benzonitrile (BN). d) Respective population kinetics. Please note that the (T_1) state was omitted in this figure, as it just begins to populate on this timescale and therefore cannot be completely deconvoluted.

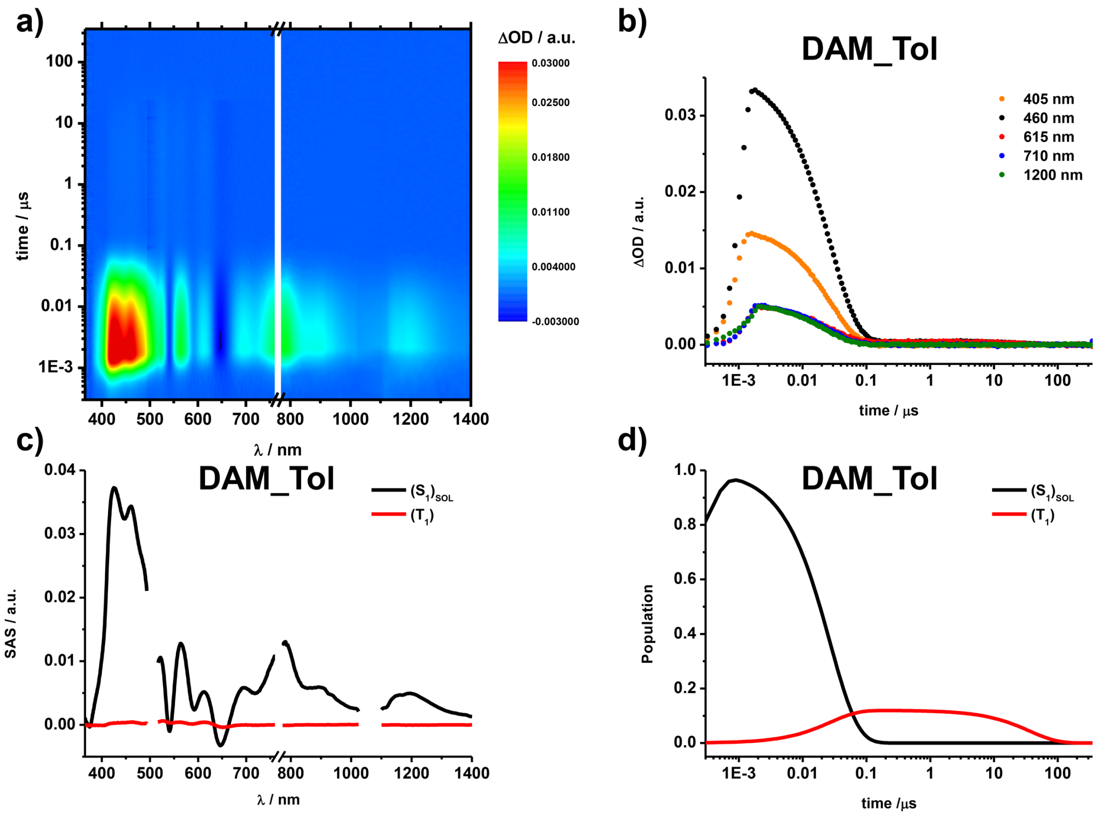

Figure S11. a) Differential ns-TA spectra (λ_{ex} = 505 nm; 500 nJ) of **DAM** in toluene (Tol) with time delays between 0 and 350 µs. b) Respective time absorption profiles at the given wavelengths. c) Deconvoluted ns-TA spectra of the stabilized singlet excited $(S_1)_{SOL}$ (black) and the triplet excited state (T_1) (red) of **DAM** as obtained by target analysis in toluene (Tol). d) Respective population kinetics.

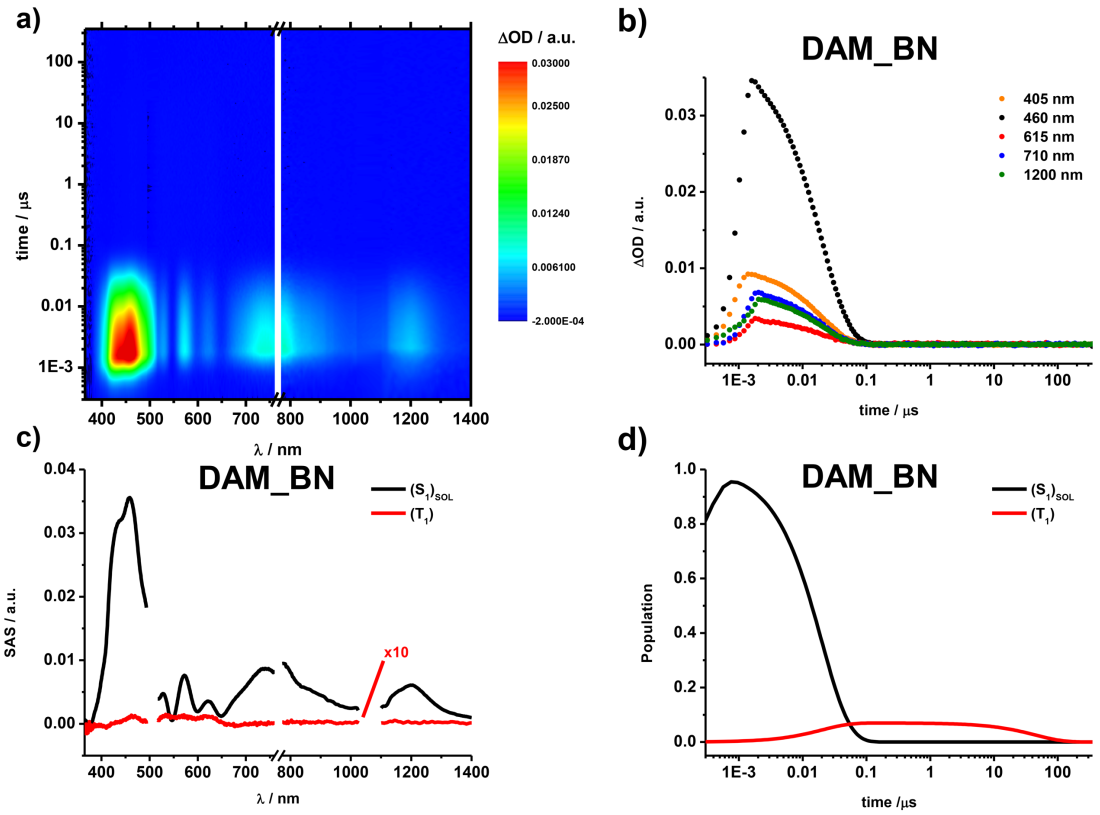

Figure S12. a) Differential ns-TA spectra (λ_{ex} = 505 nm; 500 nJ) of **DAM** in benzonitrile (BN) with time delays between 0 and 350 µs. b) Respective time absorption profiles at the given wavelengths. c) Deconvoluted ns-TA spectra of the stabilized singlet excited $(S_1)_{SOL}$ (black) and the triplet excited state (T_1) (red) of **DAM** as obtained by target analysis in benzonitrile (BN). d) Respective population kinetics.

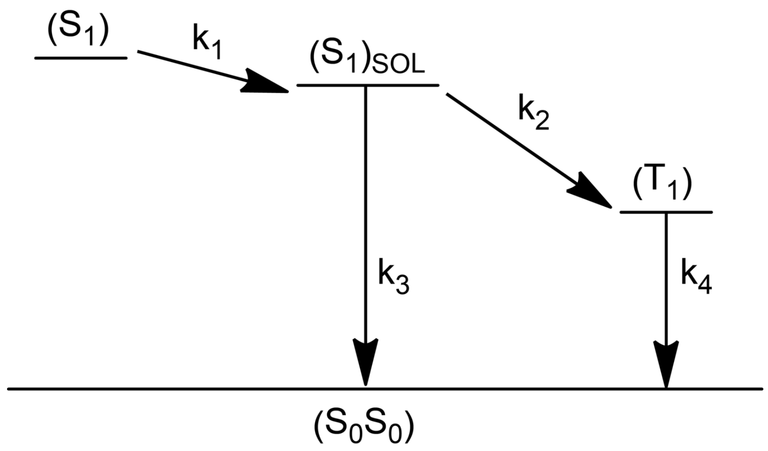

Figure S13. Kinetic model used to fit the transient absorption data for **DAM.**

Table S6. Rate constans and efficiencies of **DAM** in toluene (Tol) and benzonitrile (BN).

DAM	$k(S_1)$ / s^{-1}	$k(S_1)_{SOL}$ / s^{-1}		$k(T_1)$ / s^{-1}
**Tol**	$k_1 = 1.23 \times 10^{11}$<br>$k_1$ = 100%	$k_2 = 0.45 \times 10^7$<br>k_2 = 12%	$k_3 = 3.26 \times 10^7$<br>k_3 = 88%	$k_4 = 2.78 \times 10^4$<br>k_4 = 100%
**BN**	$k_1 = 1.05 \times 10^{11}$<br>$k_1$ = 100%	$k_2 = 0.40 \times 10^7$<br>k_2 = 8%	$k_3 = 4.60 \times 10^7$<br>k_3 = 92%	$k_4 = 2.25 \times 10^4$<br>k_4 = 100%

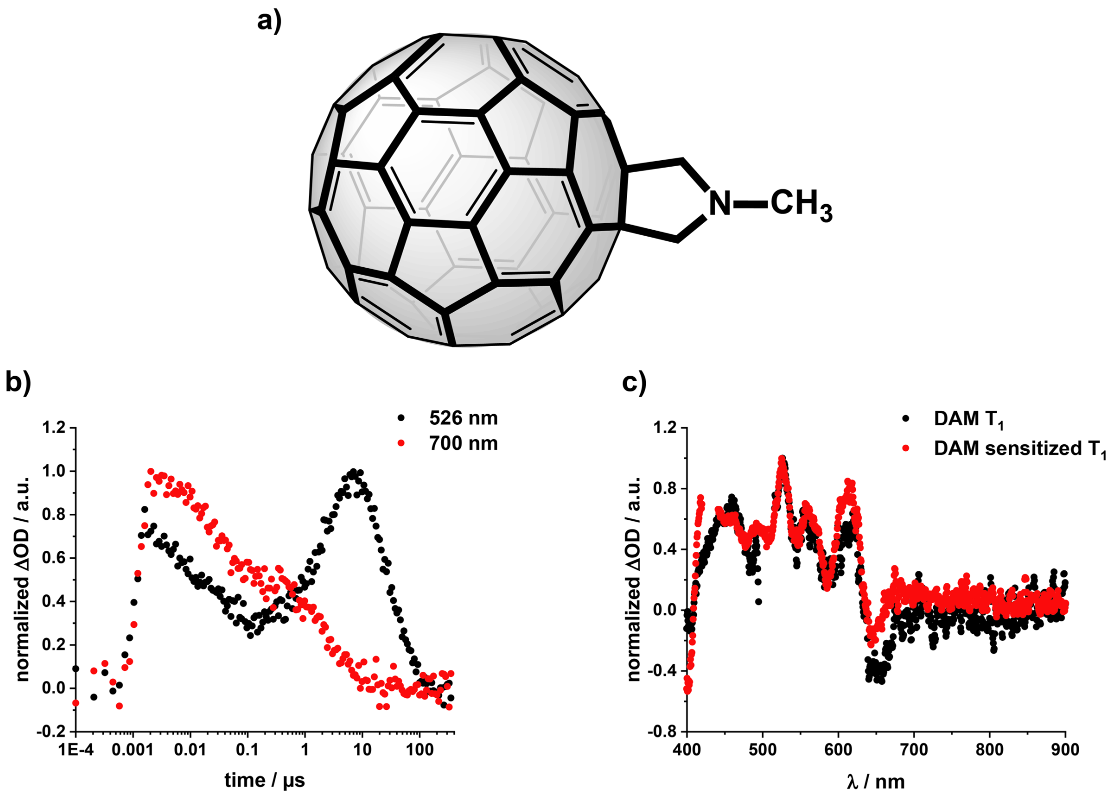

Figure S14. Triplet-triplet sensitization experiments of **DAM** (1×10^{-4} M) using a) N-methylfulleropyrrolidine(*N*-MFP) (8×10^{-5} M) as a sensitizer at 430 nm excitation (300 nJ) in argon saturated toluene at room temperature. b) Normalized time profiles of the **DAM** triplet excited state feature (526 nm; black) and the *N*-MFP triplet excited state feature (700 nm; red), illustrating the sensitization process and c) Triplet excited state spectra of **DAM** after direct excitation at 505 nm (500 nJ; black) and after sensitization with ***N*-MFP** (red).

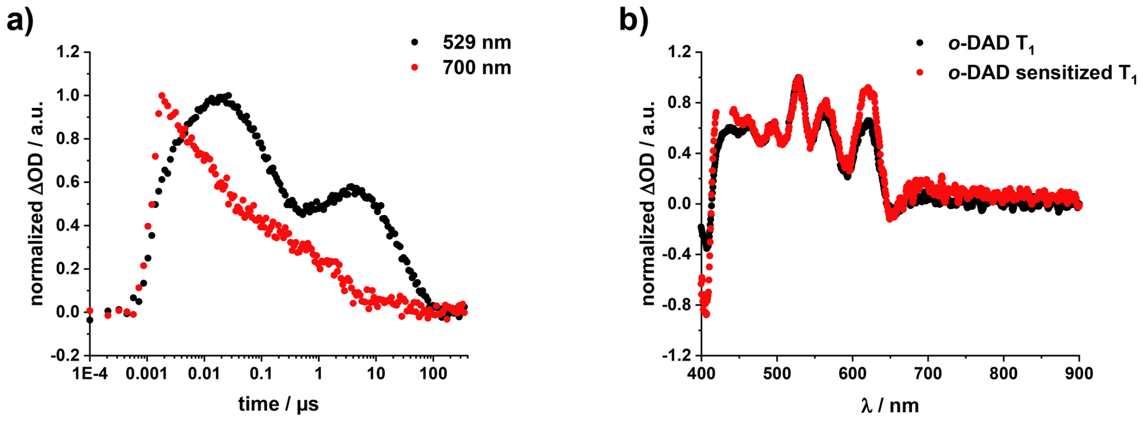

Figure S15. Triplet-triplet sensitization experiments of ***o*-DAD** (1 x 10^{-4} M) using *N*-MFP (8 x 10^{-5} M) as a sensitizer at 430 nm excitation (300 nJ) in argon saturated toluene at room temperature. a) Normalized time profiles of the ***o*-DAD** triplet excited state feature (526 nm; black) and the *N*-MFP triplet excited state feature (700 nm; red), illustrating the sensitization process and b) Triplet excited state spectra of ***o*-DAD** after direct excitation at 505 nm (500 nJ; black) and after sensitization with ***N*-MFP** (red).

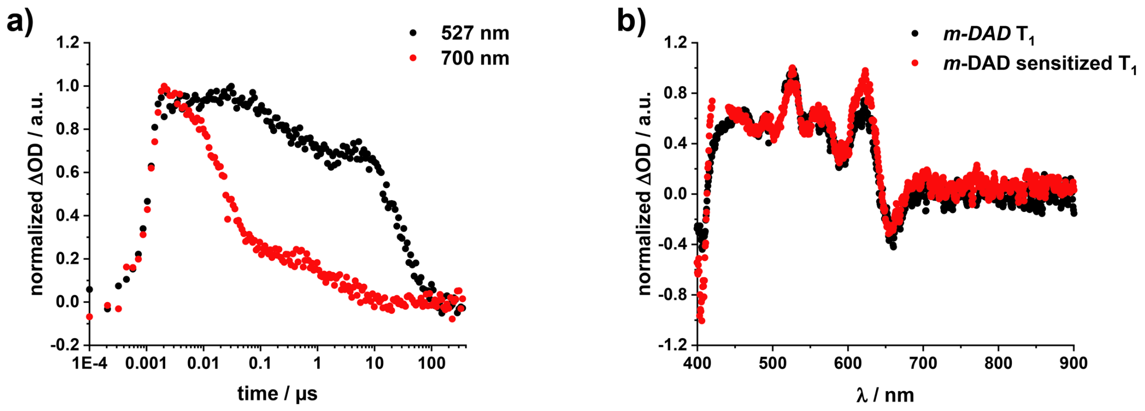

Figure S16. Triplet-triplet sensitization experiments of ***m*-DAD** (1 x 10^{-4} M) using *N*-MFP (8 x 10^{-5} M) as a sensitizer at 430 nm excitation (300 nJ) in argon saturated toluene at room temperature. a) Normalized time profiles of the ***m*-DAD** triplet excited state feature (526 nm; black) and the *N*-MFP triplet excited state feature (700 nm; red), illustrating the sensitization process and b) Triplet excited state spectra of ***m*-DAD** after direct excitation at 505 nm (500 nJ; black) and after sensitization with ***N*-MFP** (red).

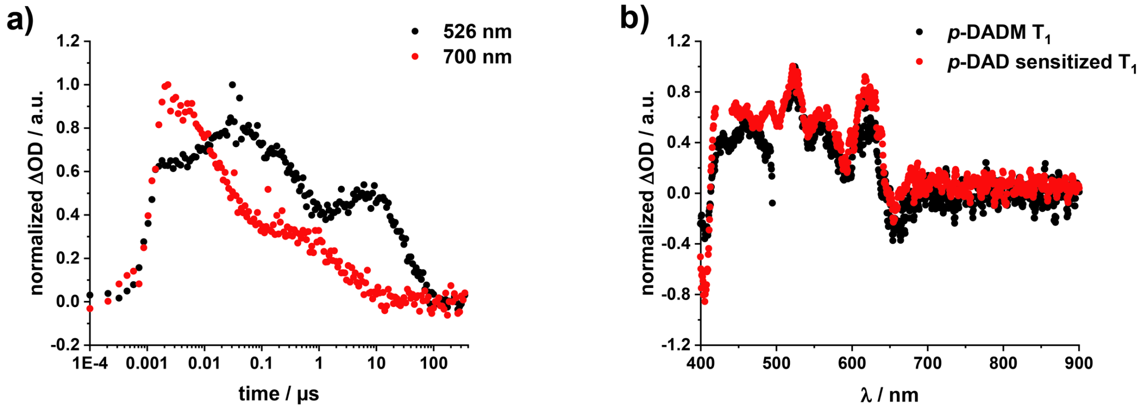

Figure S17. Triplet-triplet sensitization experiments of ***p*-DAD** (1 x 10^{-4} M) using *N*-MFP (8 x 10^{-5} M) as a sensitizer at 430 nm excitation (300 nJ) in argon saturated toluene at room temperature. a) Normalized time profiles of the ***p*-DAD** triplet excited state feature (526 nm; black) and the *N*-MFP triplet excited state feature (700 nm; red), illustrating the sensitization process and b) Triplet excited state spectra of ***p*-DAD** after direct excitation at 505 nm (500 nJ; black) and after sensitization with ***N*-MFP** (red).

Excitation at 505 nm populates the singlet excited state (S_1S_0), which undergoes solvent reorganization to afford $(S_1S_0)_{SOL}$ as first and second species, respectively. Both (S_1S_0) and $(S_1S_0)_{SOL}$ are identical to (S_1) and $(S_1)_{SOL}$ seen for **DAM**. In stark contrast to the slow ISC in **DAM**, $(S_1S_0)_{SOL}$ in ***m*-DAD** transforms quickly and directly into a correlated triplet excited $^1(T_1T_1)$ state as the third species. No intermediate is observed in the $(S_1S_0)_{SOL}$ to $^1(T_1T_1)$ transformation. The last step in the sequence is spin decoherence that results in the uncorrelated triplet excited (T_1+T_1) state as the fourth species. We infer a super-exchange mechanism, in which an energetically higher-lying CT state, that is, $(S_1S_0)_{CT}$, serves as a virtual intermediate to link $(S_1S_0)_{SOL}$ and $^1(T_1T_1)$ – vide infra. Remarkable is the detection of (T_1+T_1); in related pentacene dimers, the correlated triplet excited $^1(T_1T_1)$ state is subject to a fast and quantitative geminate triplet-triplet annihilation. The latter leads to a reinstatement of the ground state without, however, any appreciable population of the uncorrelated triplet excited (T_1+T_1) state.[4] Key to the decoherence and, in turn, (T_1+T_1) state formation are the two nitrogens, which decouples the tetracene from the phenanthrene fragment and consequently the two dibenzodiazahexacenes in ***m*-DAD**. TQYs in toluene are 70% and 27% for $^1(T_1T_1)$ and (T_1+T_1), respectively. Geminate triplet-triplet annihilation is still the major limitation in the decoherence. In benzonitrile, the TQYs of $^1(T_1T_1)$ are lower with 30% and go hand-in-hand with shorter lifetimes. Lower TQY enables corroborating a super-exchange mechanism, which is based on a virtual intermediate $(S_1S_0)_{CT}$; polar solvents stabilize CT states and, in turn, compete with the formation of $^1(T_1T_1)$. Once formed, decoherence in the $^1(T_1T_1)$ to (T_1+T_1) transformation is not impacted, as the $^1(T_1T_1)$ QY 32% is virtually identical to the findings in toluene.

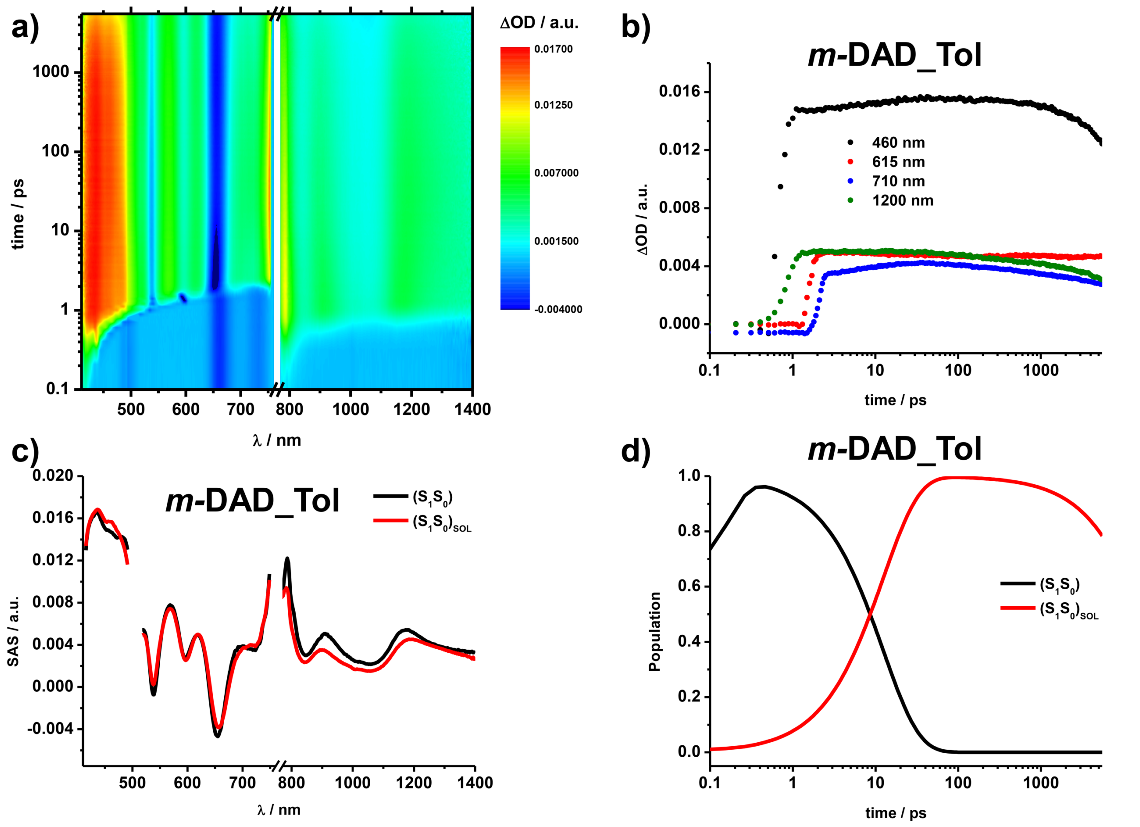

Figure S18. a) Differential fs-TA spectra (λ_{ex} = 505 nm; 500 nJ) of ***m*-DAD** in toluene (Tol) with time delays between 0 and 5 500 ps. b) Respective time absorption profiles at the given wavelengths. c) Deconvoluted fs-TA spectra of the singlet excited (S_1S_0) (black) and the stabilized singlet excited state $(S_1S_0)_{SOL}$ (red) of ***m*-DAD** as obtained by

target analysis in toluene (Tol). d) Respective population kinetics. Please note that the $^1(T_1T_1)$ state was omitted in this figure, as it just begins to populate on this timescale and therefore cannot be completely deconvoluted.

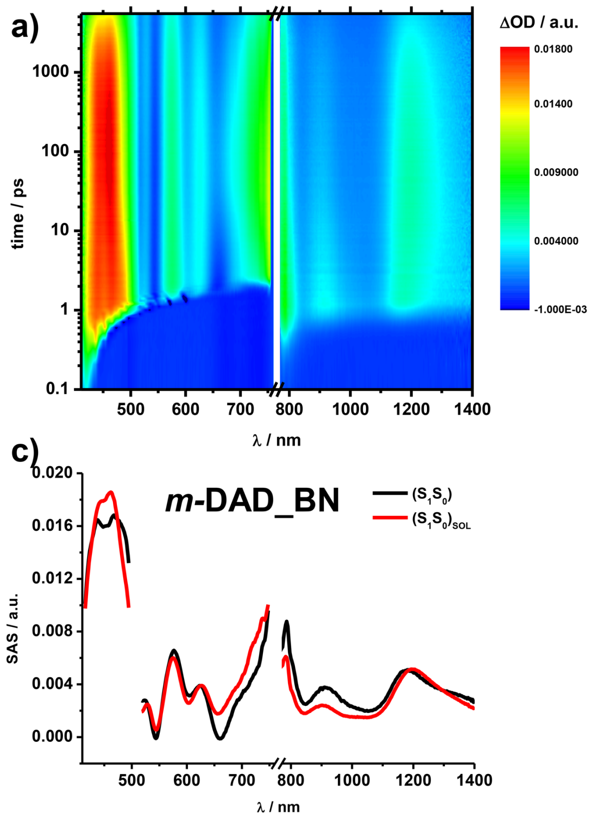

Figure S19. a) Differential fs-TA spectra (λ_{ex} = 505 nm; 500 nJ) of ***m*-DAD** in benzonitrile (BN) with time delays between 0 and 5 500 ps. b) Respective time absorption profiles at the given wavelengths. c) Deconvoluted fs-TA spectra of the singlet excited (S_1S_0) (black) and the stabilized singlet excited state $(S_1S_0)_{SOL}$ (red) of ***m*-DAD** as obtained by target analysis in benzonitrile (BN). d) Respective population kinetics. Please note that the $^1(T_1T_1)$ state was omitted in this figure, as it just begins to populate on this timescale and therefore cannot be completely deconvoluted.

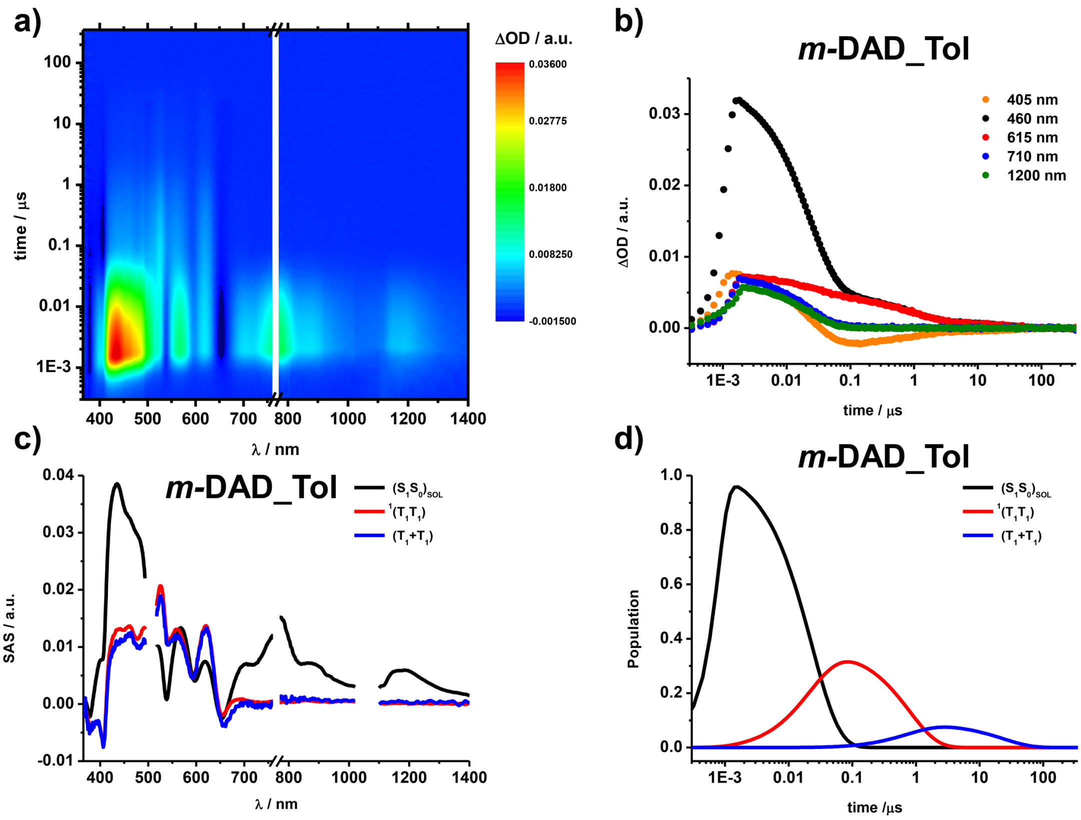

Figure S20. a) Differential ns-TA spectra (λ_{ex} = 505 nm; 500 nJ) of ***m*-DAD** in toluene (Tol) with time delays between 0 and 350 µs. b) Respective time absorption profiles at the given wavelengths. c) Deconvoluted ns-TA spectra of the stabilized singlet excited $(S_1S_0)_{SOL}$ (black), the singlet correlated triplet state $^1(T_1T_1)$ (red), and the uncorrelated triplet excited state (T_1+T_1) (blue) of ***m*-DAD** as obtained by target analysis in toluene (Tol). d) Respective population kinetics.

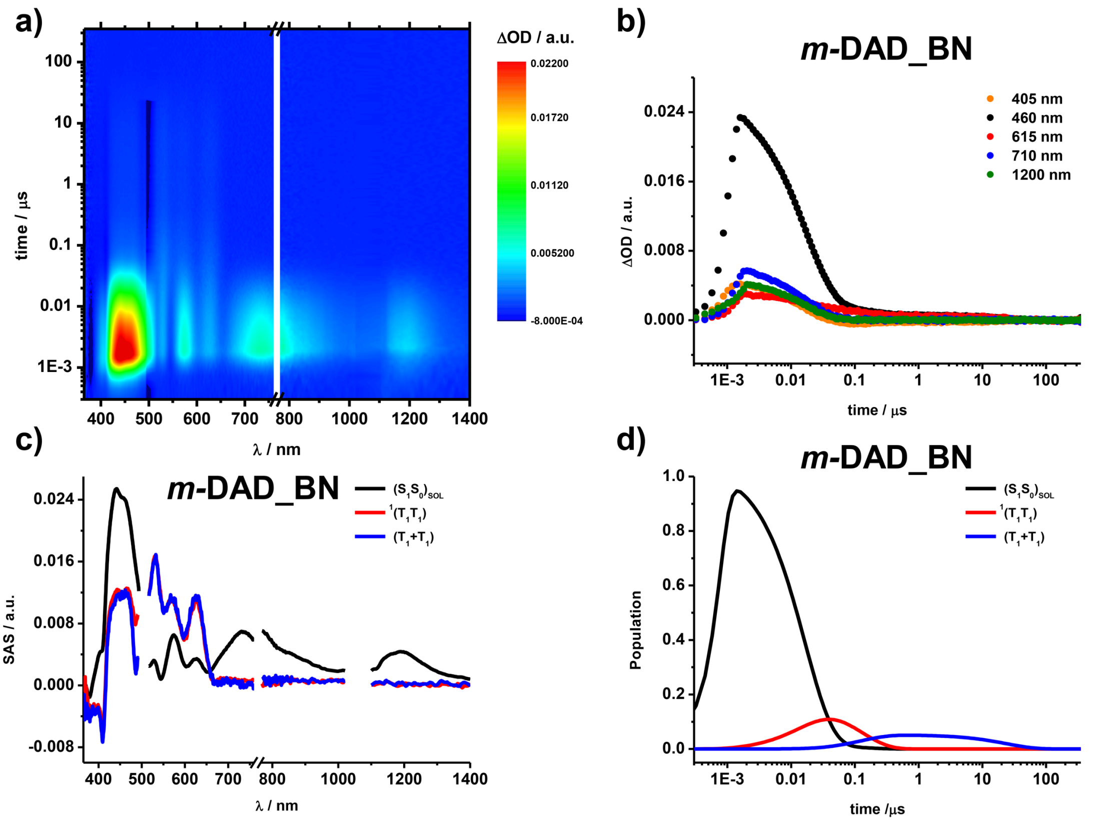

Figure S21. a) Differential ns-TA spectra (λ_{ex} = 505 nm; 500 nJ) of ***m*-DAD** in benzonitrile (BN) with time delays between 0 and 350 µs. b) Respective time absorption profiles at the given wavelengths. c) Deconvoluted ns-TA spectra of the stabilized singlet excited $(S_1S_0)_{SOL}$ (black), the singlet correlated triplet state $^1(T_1T_1)$ (red), and the uncorrelated triplet excited state (T_1+T_1) (blue) of ***m*-DAD** as obtained by target analysis in benzonitrileBN. d) Respective population kinetics.

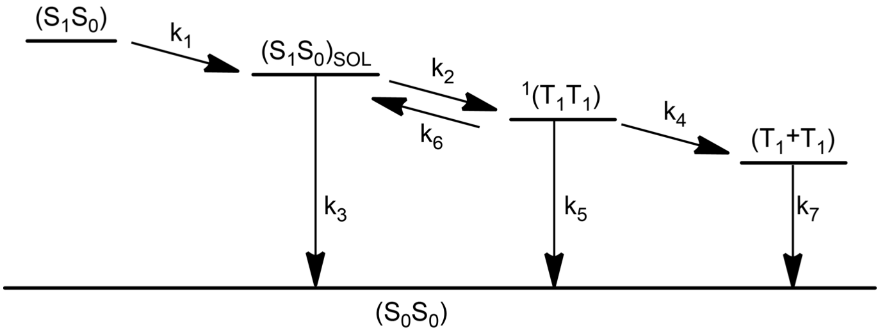

Figure S22. Kinetic model used to fit the transient absorption data for ***m*-DAD**.

Table S7. Rate constans and efficiencies of ***m*-DAD** in toluene (Tol) and benzonitrile (BN).

***m*-DAD**	**$k(S_1S_0)$ / s^{-1}**	**$k(S_1S_0)_{SOL}$ / s^{-1}**		**$k^1(T_1T_1)$ / s^{-1}**			**$k(T_1{+}T_1)$ / s^{-1}**
**Tol**	$k_1 = 8.05 \times 10^{10}$	$k_2 = 1.52 \times 10^7$	$k_3 = 2.83 \times 10^7$	$k_4 = 0.34 \times 10^6$	$k_5 = 0.86 \times 10^6$	$k_6 = 0.05 \times 10^6$	$k_7 = 4.22 \times 10^4$
	k_1 = 100%	k_2 = 35%	k_3 = 65%	k_4 = 27%	k_5 = 69%	k_6 = 4%	k_7 = 100%
**BN**	$k_1 = 9.60 \times 10^{11}$	$k_2 = 0.91 \times 10^7$	$k_3 = 5.14 \times 10^7$	$k_4 = 2.57 \times 10^6$	$k_5 = 1.45 \times 10^6$	$k_6 = 4.02 \times 10^6$	$k_7 = 4.13 \times 10^4$
	k_1 = 100%	k_2 = 15%	k_3 = 85%	k_4 = 32%	k_5 = 18%	k_6 = 50%	k_7 = 100%

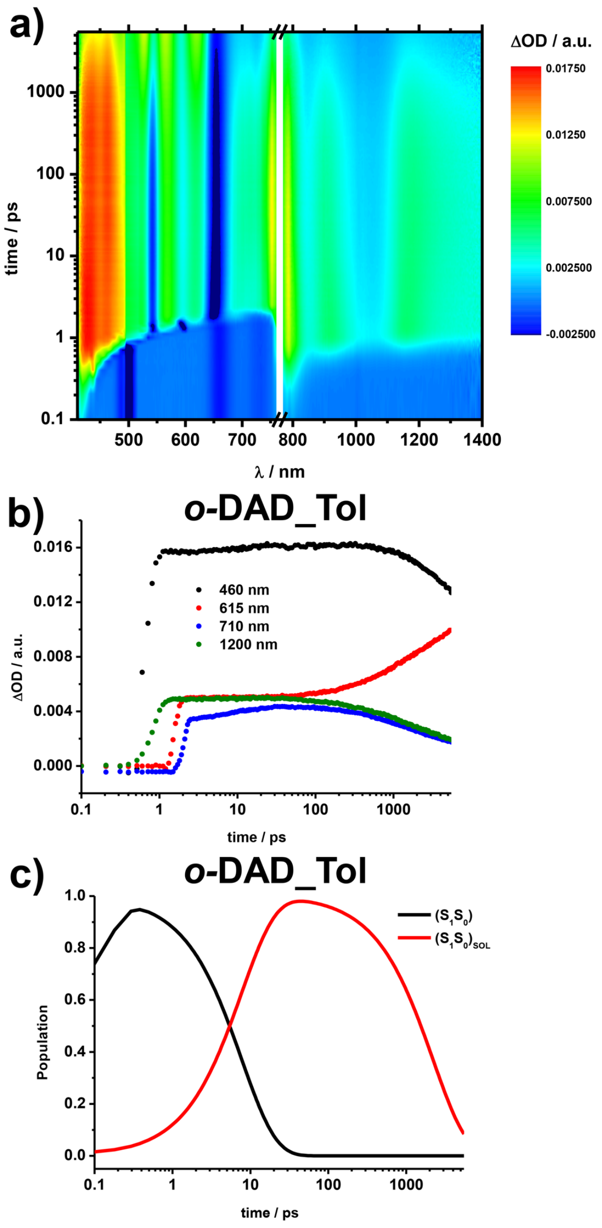

Figure S23. a) Differential fs-TA spectra (λ_{ex} = 505 nm; 500 nJ) of ***o*-DAD** in toluene (Tol) with time delays between 0 and 5 500 ps. b) Respective time absorption profiles at the given wavelengths. c) Deconvoluted fs-TA spectra of the singlet excited (S_1S_0) (black) and the stabilized singlet excited state $(S_1S_0)_{SOL}$ (red) of ***o*-DAD** as obtained by target analysis in toluene (Tol). d) Respective population kinetics. Please note that the $(S_1S_0)_{CT}$ state was omitted in this figure, as it just begins to populate on this timescale and therefore cannot be completely deconvoluted (See spectra and target analysis in toluene are shown in Figure 3).

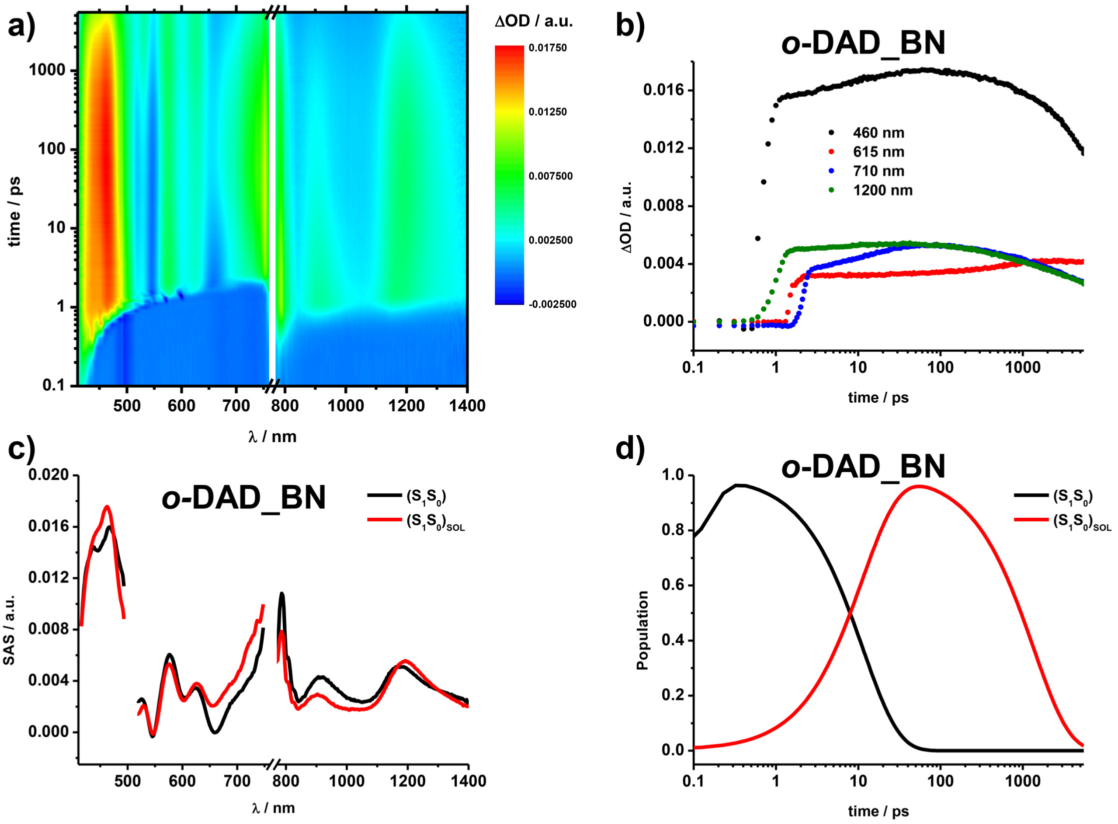

Figure S24. a) Differential fs-TA spectra (λ_{ex} = 505 nm; 500 nJ) of ***o*-DAD** in benzonitrile (BN) with time delays between 0 and 5 500 ps. b) Respective time absorption profiles at the given wavelengths. c) Deconvoluted fs-TA spectra of the singlet excited (S_1S_0) (black) and the stabilized singlet excited state $(S_1S_0)_{SOL}$ (red) of ***o*-DAD** as obtained by target analysis in benzonitrile (BN). d) Respective population kinetics. Please note that the $(S_1S_0)_{CT}$ state was omitted in this figure, as it just begins to populate on this timescale and therefore cannot be completely deconvoluted.

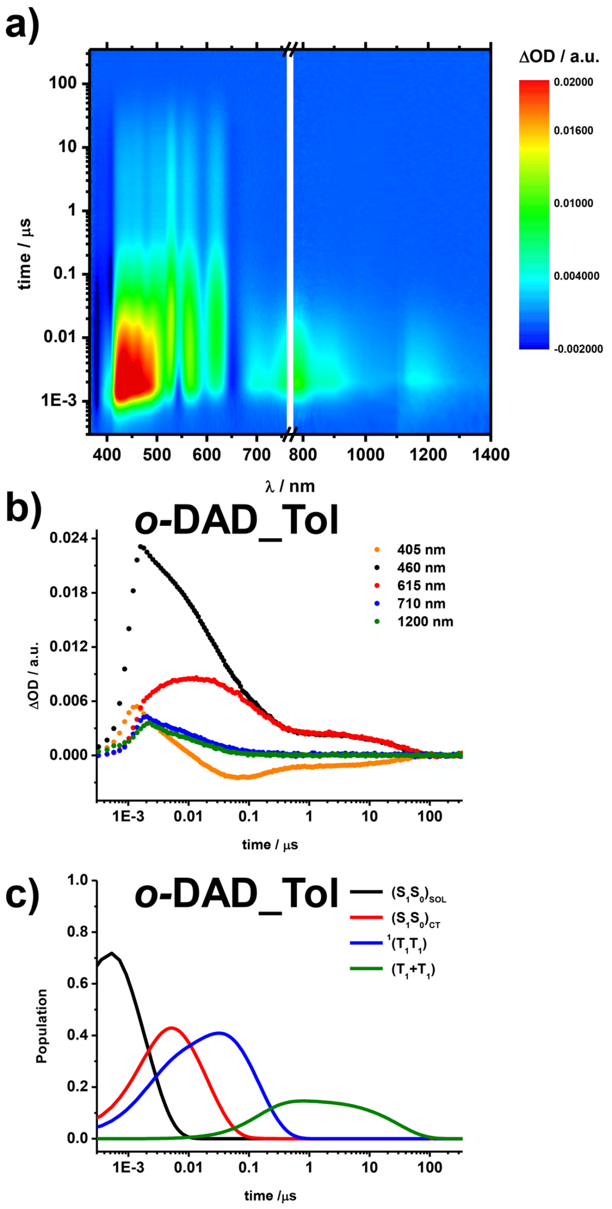

Figure S25. a) Differential ns-TA spectra (λ_{ex} = 505 nm; 500 nJ) of ***o*-DAD** in toluene (Tol) with time delays between 0 and 350 µs. b) Respective time absorption profiles at the given wavelengths. c) Deconvoluted ns-TA spectra of the stabilized singlet excited $(S_1S_0)_{SOL}$ (black), the intermediate CT-state $(S_1S_0)_{CT}$ (red), the singlet correlated triplet state $^1(T_1T_1)$ (blue), and the uncorrelated triplet excited state (T_1+T_1) (green) of ***o*-DAD** as obtained by target analysis in toluene (Tol). d) Respective population kinetics. (See spectra and target analysis in toluene are shown in Figure 3).

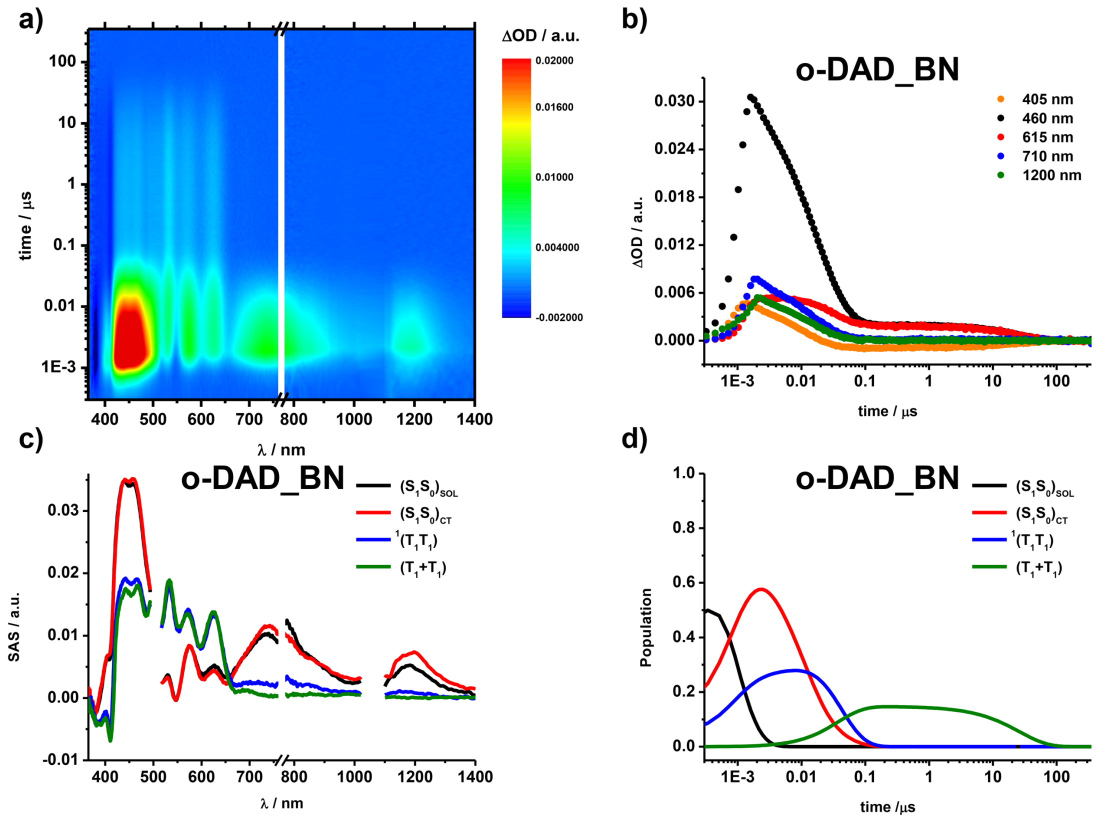

Figure S26. a) Differential ns-TA spectra (λ_{ex} = 505 nm; 500 nJ) of ***o*-DAD** in benzonitrile (BN) with time delays between 0 and 350 µs. b) Respective time absorption profiles at the given wavelengths. c) Deconvoluted ns-TA spectra of the stabilized singlet excited $(S_1S_0)_{SOL}$ (black), the intermediate CT-state $(S_1S_0)_{CT}$ (red), the singlet correlated triplet state $^1(T_1T_1)$ (blue), and the uncorrelated triplet excited state (T_1+T_1) (green) of ***o*-DAD** as obtained by target analysis in benzonitrile (BN). d) Respective population kinetics.

Table S8. Rate constans and efficiencies of ***o*-DAD** in toluene (Tol) and benzonitrile (BN).

***o*-DAD**	**$k(S_1S_0)$ / s^{-1}**	**$k(S_1S_0)_{SOL}$ / s^{-1}**			**$k(S_1S_0)_{CT}$ / s^{-1}**		**$k^1(T_1T_1)$ / s^{-1}**			**$k(T_1+T_1)$ / s^{-1}**
**Tol**	$k_1 = 1.28 \times 10^{11}$	$k_2 = 0.64 \times 10^8$	$k_3 = 0.51 \times 10^8$	$k_4 = 0.13 \times 10^8$	$k_5 = 2.17 \times 10^7$	$k_6 = 2.65 \times 10^7$	$k_7 = 2.12 \times 10^6$	$k_8 = 4.73 \times 10^6$	$k_9 = 0.21 \times 10^6$	$k_{10} = 3.27 \times 10^4$
	$k_1 = 100\%$	$k_2 = 50\%$	$k_3 = 40\%$	$k_4 = 10\%$	$k_5 = 45\%$	$k_6 = 55\%$	$k_7 = 30\%$	$k_8 = 69\%$	$k_9 = 3\%$	$k_{10} = 100\%$
**BN**	$k_1 = 1.04 \times 10^{10}$	$k_2 = 5.14 \times 10^8$	$k_3 = 1.84 \times 10^8$	$k_4 = 0.37 \times 10^8$	$k_5 = 0.91 \times 10^7$	$k_6 = 0.91 \times 10^7$	$k_7 = 0.81 \times 10^7$	$k_8 = 0.60 \times 10^7$	$k_9 = 1.58 \times 10^7$	$k_{10} = 3.80 \times 10^4$
	$k_1 = 100\%$	$k_2 = 70\%$	$k_3 = 25\%$	$k_4 = 5\%$	$k_5 = 30\%$	$k_6 = 70\%$	$k_7 = 27\%$	$k_8 = 20\%$	$k_9 = 53\%$	$k_{10} = 100\%$

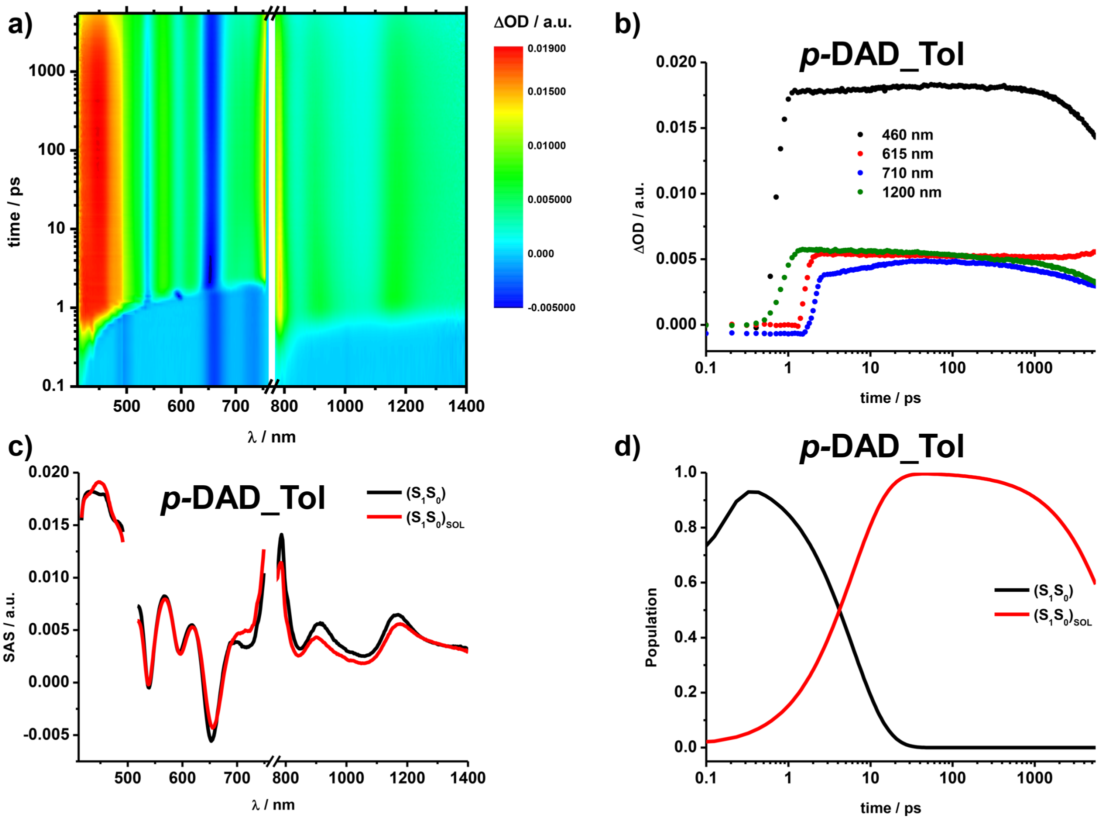

Figure S27. a) Differential fs-TA spectra (λ_{ex} = 505 nm; 500 nJ) of ***p*-DAD** in toluene (Tol) with time delays between 0 and 5 500 ps. b) Respective time absorption profiles at the given wavelengths. c) Deconvoluted fs-TA spectra of the singlet excited (S_1S_0) (black) and the stabilized singlet excited state $(S_1S_0)_{SOL}$ (red) of ***p*-DAD** as obtained by target analysis in toluene (Tol). d) Respective population kinetics. Please note that the $(S_1S_0)_{CT}$ state was omitted in this figure, as it just begins to populate on this timescale and therefore cannot be completely deconvoluted.

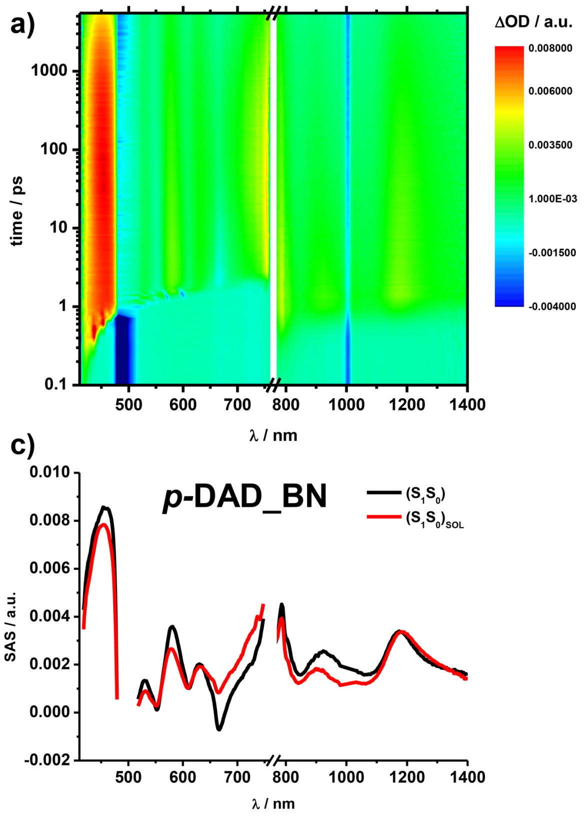

Figure S28. a) Differential fs-TA spectra (λ_{ex} = 505 nm; 500 nJ) of ***p*-DAD** in benzonitrile (BN) with time delays between 0 and 5 500 ps. b) Respective time absorption profiles at the given wavelengths. c) Deconvoluted fs-TA spectra of the singlet excited (S_1S_0) (black) and the stabilized singlet excited state $(S_1S_0)_{SOL}$ (red) of ***p*-DAD** as obtained by target analysis in benzonitrile (BN). d) Respective population kinetics. Please note that the $(S_1S_0)_{CT}$ state was omitted in this figure, as it just begins to populate on this timescale and therefore cannot be completely deconvoluted.

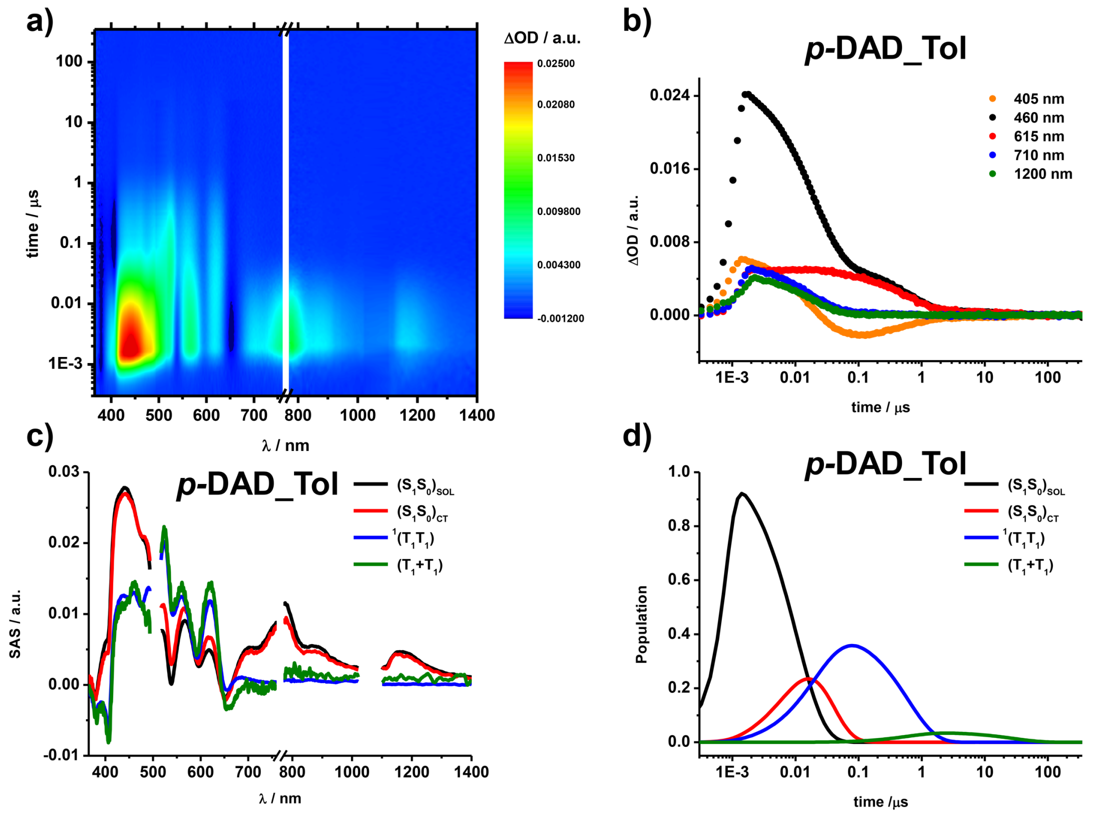

Figure S29. a) Differential ns-TA spectra (λ_{ex} = 505 nm; 500 nJ) of ***p*-DAD** in toluene (Tol) with time delays between 0 and 350 μs. b) Respective time absorption profiles at the given wavelengths. c) Deconvoluted ns-TA spectra of the stabilized singlet excited $(S_1S_0)_{SOL}$ (black), the intermediate CT-state $(S_1S_0)_{CT}$ (red), the singlet correlated triplet state $^1(T_1T_1)$ (blue), and the uncorrelated triplet excited state (T_1+T_1) (green) of ***p*-DAD** as obtained by target analysis in toluene (Tol). d) Respective population kinetics.

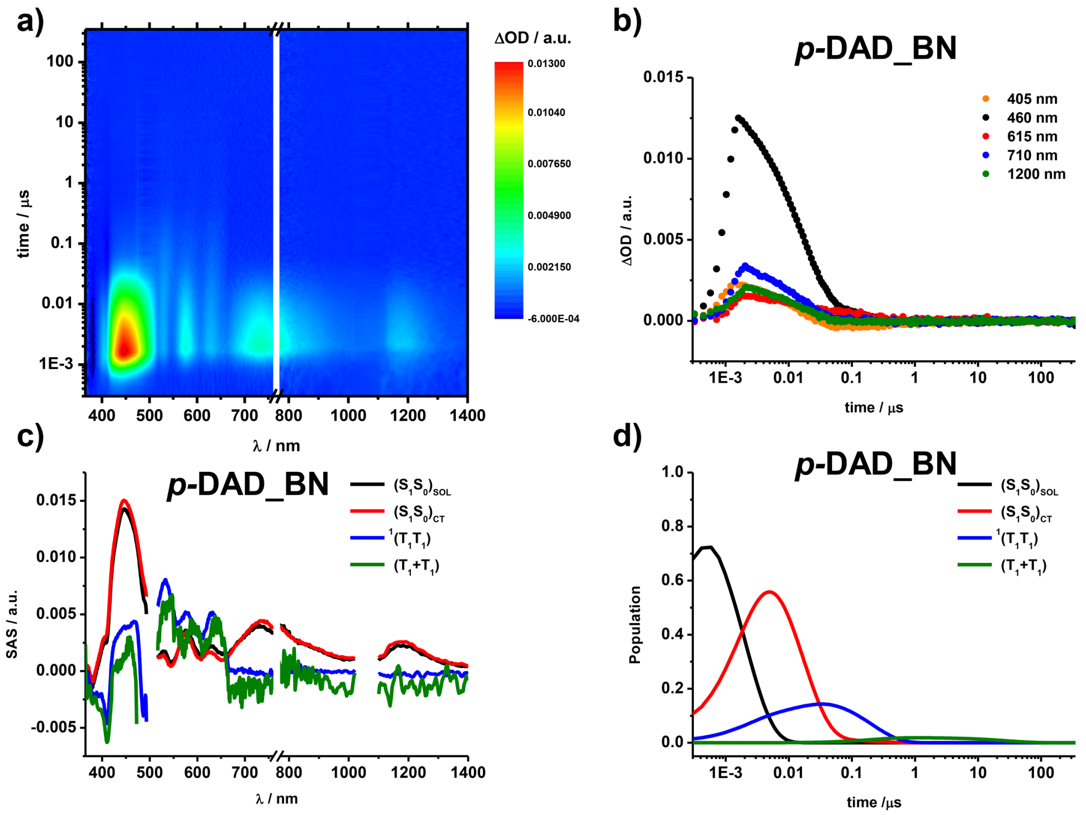

Figure S30. a) Differential ns-TA spectra (λ_{ex} = 505 nm; 500 nJ) of ***p*-DAD** in benzonitrile (BN) with time delays between 0 and 350 µs. b) Respective time absorption profiles at the given wavelengths. c) Deconvoluted ns-TA spectra of the stabilized singlet excited $(S_1S_0)_{SOL}$ (black), the intermediate CT-state $(S_1S_0)_{CT}$ (red), the singlet correlated triplet state $^1(T_1T_1)$ (blue), and the uncorrelated triplet excited state (T_1+T_1) (green) of ***p*-DAD** as obtained by target analysis in benzonitrile (BN). d) Respective population kinetics.

Table S9. Rate constans and efficiencies of ***p*-DAD** in toluene (Tol) and benzonitrile (BN).

***p*-DAD**	**k(S_1S_0) / s^{-1}**	**k(S_1S_0)$_{SOL}$ / s^{-1}**			**k(S_1S_0)$_{CT}$ / s^{-1}**		**k^1(T_1T_1) / s^{-1}**			**k(T_1+T_1) / s^{-1}**
**Tol**	k_1 = 7.80 x 10^{10}	k_2 = 4.27 x 10^7	k_3 = 1.90 x 10^7	k_4 = 3.32 x 10^7	k_5 = 1.95 x 10^7	k_6 = 2.29 x 10^7	k_7 = 0.16 x 10^6	k_8 = 1.44 x 10^6	k_9 = 0.02 x 10^6	k_{10} = 3.45 x 10^4
	k_1 = 100%	k_2 = 45%	k_3 = 20%	k_4 = 35%	k_5 = 46%	k_6 = 54%	k_7 = 10%	k_8 = 89%	k_9 = 1%	k_{10} = 100%
**BN**	k_1 = 9.83 x 10^{10}	k_2 = 3.68 x 10^8	k_3 = 0.49 x 10^8	k_4 = 0.73 x 10^8	k_5 = 0.61 x 10^7	k_6 = 5.51 x 10^7	k_7 = 0.47 x 10^6	k_8 = 0.24 x 10^6	k_9 = 4.02 x 10^6	k_{10} = 3.46 x 10^4
	k_1 = 100%	k_2 = 75%	k_3 = 10%	k_4 = 15%	k_5 = 10%	k_6 = 90%	k_7 = 10%	k_8 = 5%	k_9 = 85%	k_{10} = 100%

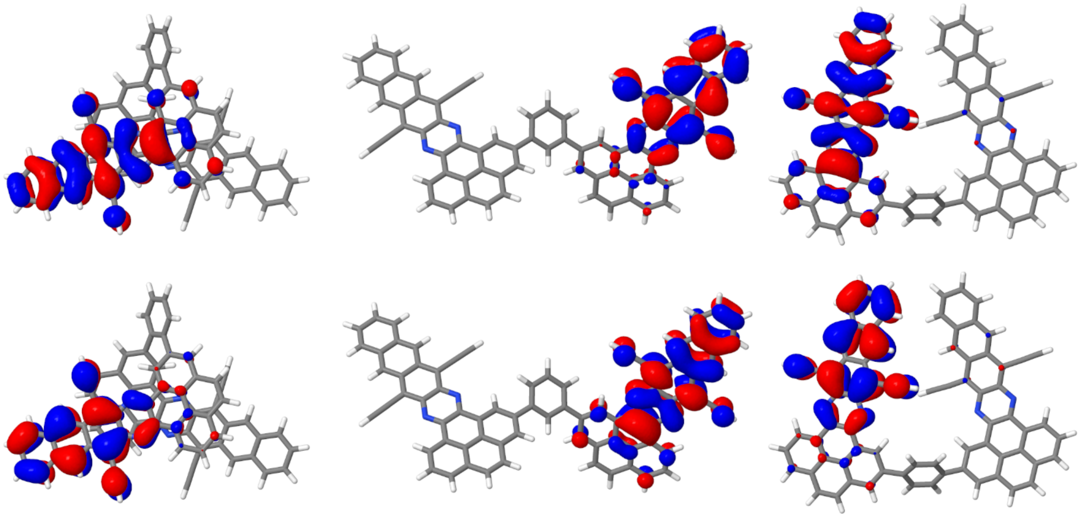

$S_0 \rightarrow S_1$

Figure S31. Electrons (top) and holes (bottom) for the $S_0 \rightarrow S_1$ transition of, from left to right ***o*-DAD-H**, ***m*-DAD-H** and ***p*-DAD-H** model molecules (this is a model system in which the TIPS and tert-butyl groups have been exchanged by H) at the M06-2X-toluene-6-311+g(2d,p)/PBEh-3c level from NTO analysis. $S_0 \rightarrow S_{1'}$ transitions are basically equivalent.

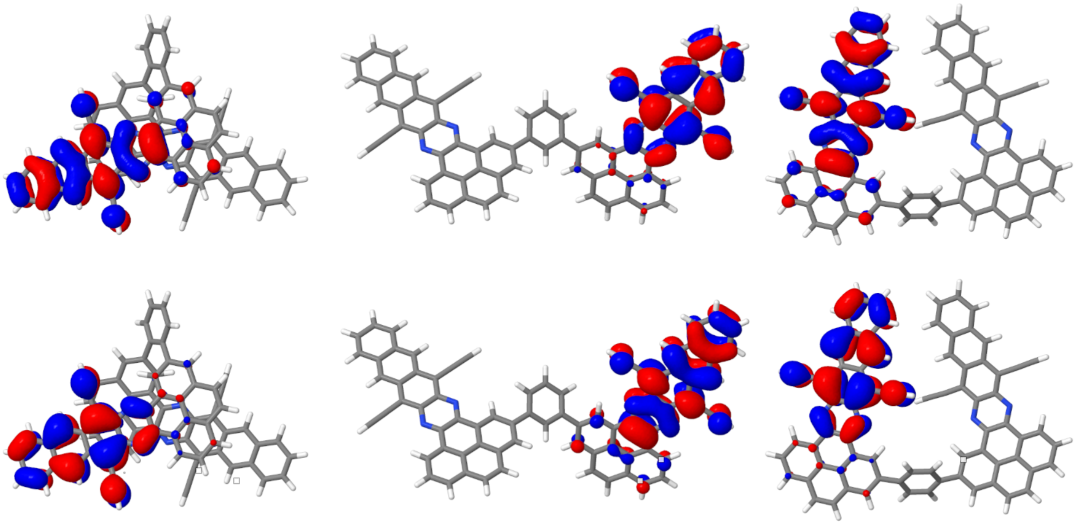

$S_0 \rightarrow T_1$

Figure S32. Electrons (top) and holes (bottom) for the $S_0 \rightarrow T_1$ dark transition of, from left to right ***o*-DAD-H**, ***m*-DAD-H** and ***p*-DAD-H** model molecules (this is a model system in which the TIPS and tert-butyl groups have been exchanged by H) at the M06-2X-toluene-6-311+g(2d,p)/PBEh-3c level from NTO analysis. $S_0 \rightarrow T_{1'}$ transitions are basically equivalent.

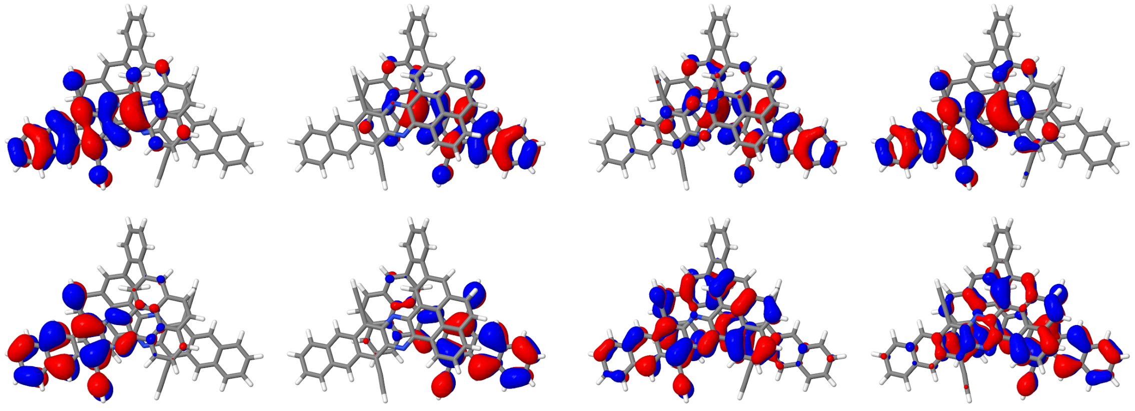

$S_0 \rightarrow S_1$	$S_0 \rightarrow S_{1'}$	$S_0 \rightarrow S_2$	$S_0 \rightarrow S_{2'}$
**2.09**	**2.10**	**2.60**	**2.65**
**(LE)**	**(LE)**	**(CT)**	**(CT)**

Figure S33. Electrons (top) and holes (bottom) for the first four transitions of the ***o*-DAD-H** model molecule (this is a model system in which the TIPS and tert-butyl groups have been exchanged by H) at the M06-2X-toluene-6-311+g(2d,p)/PBEh-3c level from NTO analysis showing Local Excitations (LE) and Charge Transfer excitations (CT).

Table S10. Transition energies and oscillator strengths of the first four transitions of ***o*-DAD-H** model (this is a model system in which the TIPS and tert-butyl groups have been exchanged by H) molecule computed with NEVPT2(8,8)/def-SV(P) and M06-2X-toluene-6-311+g(2d,p)/PBEh-3c level with different character: Local Excitations (LE) and Charge Transfer excitations (CT).

	NEVPT2(8,8)/def-SV(P)		M06-2X-Toluene- 6-311g+g(2d,p)/PBEh-3c	
	Energy / eV	osc. / U.A.	Energy / eV	osc. / U.A.
S_1 (LE)	2.28	0.107	2.09	0.04
$S_{1'}$ (LE)	2.39	0.124	2.10	0.15
S_2 (CT)	2.36	0.057	2.60	0.22
$S_{2'}$ (CT)	2.37	0.026	2.65	0.08

Experimental Procedures

Synthesis and characterization

Commercial chemicals and solvents were used as received.

Analytical thin layer chromatography (TLC) was carried out using aluminum sheets (20x20 cm) pre-coated with silica gel RP-18W 60 F254 from Merck.

Column chromatography was carried out using Silica gel 60 (40-60 µm) from Scharlab.

NMR spectra in solution were recorded on a Bruker Avance 400 MHz or 500 MHz spectrometer at 298 K using partially deuterated solvents as internal standards.

High Resolution Matrix Assisted Laser Desorption Ionization (coupled to a Time-Of-Flight analyzer) Mass Spectrometry experiments were recorded in Biomagune in a Ultraflex III (Bruker Daltonics) MALDI-ToF (frequency-tripled (355 nm) Nd:YAG laser) by Dr. Javier Calvo. Matrix Assisted Laser Desorption Ionization (coupled to a Time-Of-Flight analyzer) experiments MALDI-TOF) were recorded on Bruker REFLEX spectrometer in POLYMAT by Dr. Estíbaliz González de San Román Martín.

Thermal gravimetric analysis carried out on a Mettler Toledo TGA /SDTA 851 in a sealed in an aluminium pan, and measured at a heating rate of 10 ºC min^{-1} under a nitrogen flow.

X-ray single crystal diffraction experiments were performed by the X-ray diffraction unit of General Services SG-Iker (UPV/EHU) by Dr. Leire San Felices. Intensity data were collected on an Agilent Technologies Super-Nova diffractometer, which was equipped with monochromated Cu kα radiation (λ= 1.54184 Å) and Atlas CCD detector. Measurement was carried out at 150.00(10) K with the help of an Oxford Cryostream 700 PLUS temperature device. Data frames were processed (united cell determination, analytical absorption correction with face indexing, intensity data integration and correction for Lorentz and polarization effects) using the Crysalis software package. The structure was solved using Olex2 and refined by full-matrix least-squares with SHELXL-97. Final geometrical calculations were carried out with Mercury and PLATON as integrated in WinGX.

Steady-state electronic absorption

Absorption spectra were recorded on a Perkin-Elmer Lambda 950 spectrometer.

Photoluminescence

Photoluminescence spectra were recorded on a LS55 Perkin-Elmer Fluorescence spectrometer.

Electrochemistry

Electrochemical measurements were carried out on a Princeton Applied Research Parstat 2273 in a 3-electrode single compartment cell with glassy carbon disc working electrode, a platinum wire counter electrode and a silver wire pseudoreference electrode. All the potential values are reported versus the redox potential of the ferrocene/ferrocenium couple.

Calculations

The generation of conformers was done at the xtb-GFN2 level with a simulated annealing procedure comprising three Molecular Dynamics steps of 50 ps each between 298 K and 1000 K. It is of note, that when compared the three dimer molecules, the ortho connection constrains strongly the possible conformations so that all the simulated annealing conformations for o-DAD yielded very similar conformations. The lowest energy minima were reoptimized with the composite method based on the PBE hybrid functional proposed by Grimme (PBEh-3c) level.[5] After, the electronic properties, computed with the B3LYP Hamiltonian in vacuum with the 6-311+g(2d,p) basis set with the computation of the spectra at the 6-31+g(d,p) level. Two models were considered, the full model, where electronic properties were computed with the B3LYP Hamiltonian in vacuum with the 6-311+g(2d,p) basis set, and a reduced model (this is a model system in which the TIPS and tert-butyl groups have been exchanged by H) that were optimized at the B3LYP-6-31g(d,p) level with all heavier atoms fixed. For these ‘cut’ systems, different basis sets were tried in toluene and vacuum with the M06-2X and B3LYP Hamiltonians. In addition, we computed the vertical excitation energies for ***o*-DAD** computed at the NEVPT2(8,8)/def-SV(P) theory level with the auxiliary basis set def2/JK basis set in conjunction with the RI-JK approximation for 20 states: 10 singlets and 10 triplets. Orca 4.1.0 was used for the CASSCF and PBEh-3c,[6] while the other DFT models were computed with Gaussian 09.

Time-correlated single photon counting

Fluorescence lifetimes were determined by the time-correlated single photon counting (TCSPC) technique using a FluoroLog3 emission spectrometer (Horiba JobinYvon) equipped with an R3809U-58 MCP (Hamamatsu) and an EXW-6 (NKT) SuperK Extreme highpower supercontinuum fiber laser with excitation at 505 nm (150 ps fwhm).

Transient absorption spectroscopy

Femtosecond transient absorption (TA) experiments were carried out with an amplified Ti:sapphire CPA-2110 fs laser system (Clark MXR: output 775 nm, 1 kHz, 150 fs pulse width) using transient absorption pump/probe detection systems (Helios and Eos, Ultrafast Systems) with argonpurged solutions. The 480 and 505 nm excitation wavelengths, with energies of 800 and 400 nJ, respectively, were generated with a noncolinear optical parametric amplifier (NOPA, Clark MXR).

Data evaluation of the fs- and ns-TAS data has been conducted by means of multiwavelength and Glotaran target analysis. Glotaran target analysis was performed on the TA data sets using the proposed kinetic models. The analytical solution to the coupled differential equations that describe the kinetic model is convoluted with a Gaussian instrument response function. After the least-squares fitting has converged, the raw data matrix is deconvoluted using the specific solution to the kinetic model and parameters from the fit to obtain the species-associated spectra and their populations as a function of time.

Triplet quantum yield (TQY) determination

The TQY determination was performed by using the normalization of the spectra during the ground state bleaching using Glotaran target analysis as found in the literature.[1b, 2] In short, this approach is based on certain assumptions: (1) the singlet excited state is delocalized over the entire molecule, thus bleaching both dibenzodiazahexacene units; (2) a single triplet excited state likewise bleaches the entire ground state singlet transition; (3) no overlying/additional features are located in the ground state bleaching area, in this case at roughly 380 nm, which allows one to normalize the triplet excited state spectra relative to the singlet excited state ones; and (4) two triplet excited states on the dimer lead to a bleaching twice that of a single one. By combining these criteria and the kinetic model in a Glotaran target analysis, one is able to determine the efficiency and rate constant of each transition. In the context of the TQY, all transitions prior to the formation of $^{1}(T_1T_1)$ contribute to its overall yield.

Synthesis of the compound A

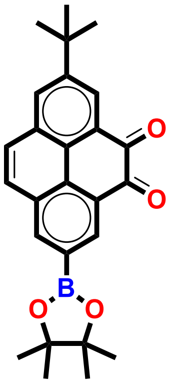

To a solution of 2-(7-(tert-butyl)pyren-2-yl)-4,4,5,5-tetramethyl-1,3,2-dioxaborolane[7] (200 mg 0.52 mmol) in CH_2Cl_2 (8 mL) and CH_3CN (8 mL) were added $NaIO_4$ (0.52 g, 2.45 mmol), H_2O (15.0 mL), and $RuCl_3$,xH_2O (10.7 mg, 0.052mmol). The dark brown suspension was stirred at room temperature overnight. The reaction mixture was poured into 100 mL of H_2O and the organic phase was extracted with CH_2Cl_2 giving an orange solution. The solvent was removed under reduced pressure to afford an orange solid. The crude was purified by chromatography column using a mixture of solvent hexane:chloroform (1:4). The orange solid was precipitated in a mixture of solvents dichloromethane and methanol, the product was obtained pure as bright orange solid (52 mg, 24 %)

^{1}H NMR (400 MHz, $CDCl_3$): 8.85 (d, J = 1.2 Hz, 1H), 8.58 (dd, J = 5.2, 1.7 Hz, 2H), 8.14 (d, J = 2.0 Hz, 1H), 7.82 (d, J = 4.2 Hz, 2H), 1.49 (s, 9H), 1.42 (s, 12H); ^{13}C-NMR (101 MHz, $CDCl_3$): ^{13}C NMR (101 MHz, $CDCl_3$) 180.96, 152.07, 142.87, 135.90, 132.76, 131.93, 131.22, 130.33, 130.14, 129.19, 128.69, 127.57, 127.54, 126.39, 84.71, 35.44, 31.31, 25.09. MS (MALDI, pos.) (m/z) $[M+Na]^+$: Calcd $C_{26}H_{27}BNaO_4$: 437.190, found: 437.169.

.

Synthesis of the compound DAM

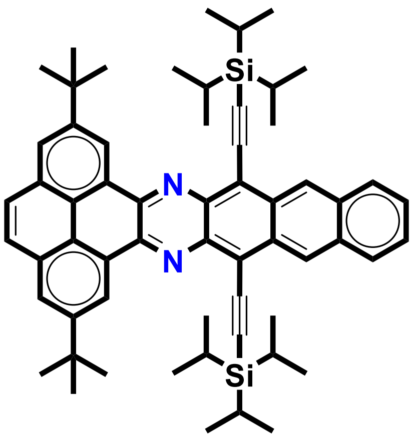

2,7-Di-*tert*-butyl pyrene-4,5-dione (25 mg, 0.072 mmol) and compound **D** (82 mg, 0.1451 mmol) were dissolved in chloroform and acetic acid (3:1) and the reaction was refluxed for 48 hours. Water was added and the product was extracted with CH_2Cl_2 (3x25). The organic phase was dried over sodium sulfate, filtrated and eliminated by rotary evaporation. The crude was loaded onto a chromatographic column (eluent : mixture hexane : dichloromethane 8:2). The resulting solids were precipitations repeatedly until the product was obtained pure as bright purple solid (40 mg, yield of 61%).

^{1}H NMR (400 MHz, CD_2Cl_2): 9.75 (d, J = 1.9 Hz, 2H), 9.60 (s, 2H), 8.30 (d, J = 1.9 Hz, 2H), 8.16 – 8.08 (m, 2H), 8.02 (s, 2H), 7.61 – 7.53 (m, 2H), 1.66 (s, 18H), 1.43 (d, J = 6.8 Hz, 42H). ^{13}C NMR (101 MHz, CD_2Cl_2): 156.05, 150.42, 146.13, 139.96, 133.09, 132.80, 131.72, 129.49, 128.84, 127.65, 127.24, 127.12, 125.08, 123.40, 120.67, 108.65, 104.22, 35.73, 31.91, 19.33, 12.20. MS (MALDI, pos.) (*m/z*): Calcd $C_{60}H_{72}N_2Si_2$: 876.523 $[M]^+$, found : 876.534.

Synthesis of the compound E

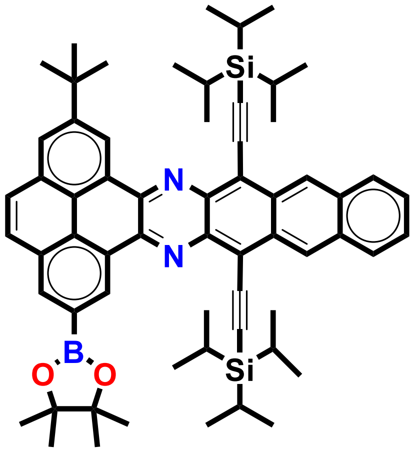

Compound **A** (25 mg, 0.06mmol) and compound **D**[8] (78 mg, 0.12 mmol) were dissolved in chloroform and acetic acid (3:1) and the reaction was refluxed for 48 hours. Water was added and the product was extracted with chloroform (3x25 mL). The organic phase was dried over sodium sulfate, filtrated and eliminated by rotary evaporation. The crude was loaded onto a chromatographic column (eluent: mixture hexane:dichloromethane 8:2). The resulting solids were precipitations repeatedly until the product was obtained pure as bright purple solid (91 mg, 56 %).

^{1}H NMR (400 MHz, CD_2Cl_2): 9.91 (s, 1H), 9.78 (d, 1H), 9.58 (d, 2H), 8.77 (s, 1H), 8.33 (d, J = 1.8 Hz, 1H), 8.16 – 8.01 (m, 4H), 7.62 – 7.53 (m, 2H), 1.67 (s, 9H), 1.54 – 1.40 (m, 54H); ^{13}C-NMR (101 MHz, CD_2Cl_2): ^{13}C NMR (101 MHz, CD_2Cl_2): 151.52, 146.37, 146.06, 140.52, 140.15, 138.52, 133.34, 133.27, 133.04, 132.78, 132.67, 131.19, 130.82, 130.32, 129.34, 129.08, 129.06, 128.95, 128.00, 127.95, 127.52, 127.45, 127.38, 127.31, 125.21, 123.68, 121.05, 120.70, 109.17, 108.79, 104.41, 84.79, 48.96, 36.00, 32.07, 25.39, 19.59, 19.53, 12.40, 12.18.MS (MALDI, pos.) (*m/z*): Calcd $C_{62}H_{75}BN_2O_2Si_2$: 946.546 $[M]^{+}$, found: 946.555.

Synthesis of the compound *m*-DAD

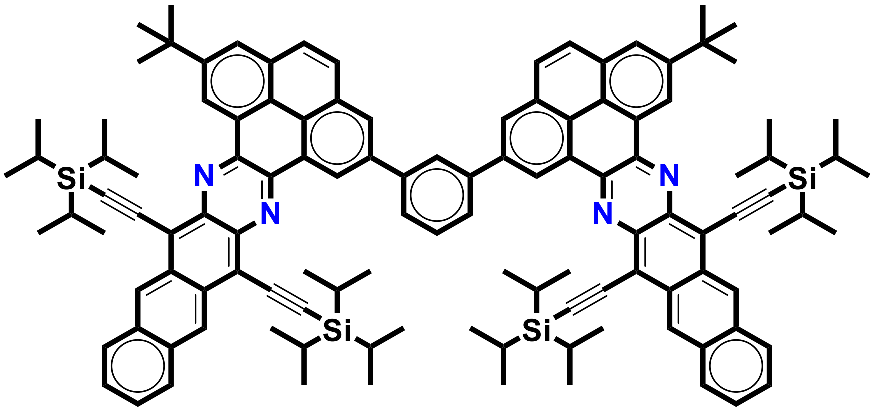

A solution of 1,3-diiodobenzene (15 mg, 0.0454 mmol) and the compound **E** (100 mg, 0.109 mmol), $PdCl_2$(dppf) (4 mg, 0.005 mol) as a catalyst, K_3PO_4 as the base (48 mg, 0.227 mmol)7 and THF/water (3 : 1 v/v) as the reaction medium. At the pulsed microwave power level of 100 W (80 °C, 120 PSI, 11 min). The product was extracted with CH_2Cl_2 (3 x 30 mL), dried over sodium sulfate and the organic phase was removed by rotary evaporation. The resulting solids were loaded onto a chromatographic column (eluent: mixture hexane : dichloromethane 8:2). The resulting solids were precipitated until obtain the desired product as bright purple solid (35 mg, 45 %).

^{1}H NMR (400 MHz,CD_2Cl_2): 10.00 (d, *J* = 1.8 Hz, 2H), 9.84 (d, *J* = 1.9 Hz, 2H), 9.64 (s, *J* = 8.0 Hz, 2H), 9.55 (s, 2H), 8.65 (d, *J* = 1.7 Hz, 2H), 8.49 (s, 1H), 8.36 (d, *J* = 2.0 Hz, 2H), 8.22 – 8.06 (m, 10H), 7.88 (t, J = 7.6 Hz, 1H), 7.59 (m, J = 6.8, 3.3 Hz, 4H), 1.72 (s, 18H), 1.58 – 1.40 (m, 42H), 1.26 – 1.10 (m, 42H); ^{13}C NMR (101 MHz, CD_2Cl_2): 150.89, 145.96, 145.84, 142.35, 140.48, 140.12, 140.00, 133.14, 133.05, 132.88, 132.59, 132.33, 131.98, 130.43, 129.93, 129.77, 129.70, 128.84, 128.33, 127.86, 127.62, 127.36, 127.36, 127.20, 127.13, 126.30, 125.03, 124.28, 123.61, 120.92, 120.57, 108.91, 108.69, 104.20, 104.00, 83.24, 35.78, 31.90, 19.33, 19.06, 12.20, 11.88. MS (MALDI): Calcd $C_{118}H_{131}N_4Si_4$: $[M+H]^+$:1715.9442 Found $[M+H]^+$:1715.9418. MS (MALDI): Calcd $C_{118}H_{130}AgN_4Si_4$: $[M+Ag]^+$:1821.8415 Found $[M+Ag]^+$:1821.8416.

Synthesis of the compound *o*-DAD

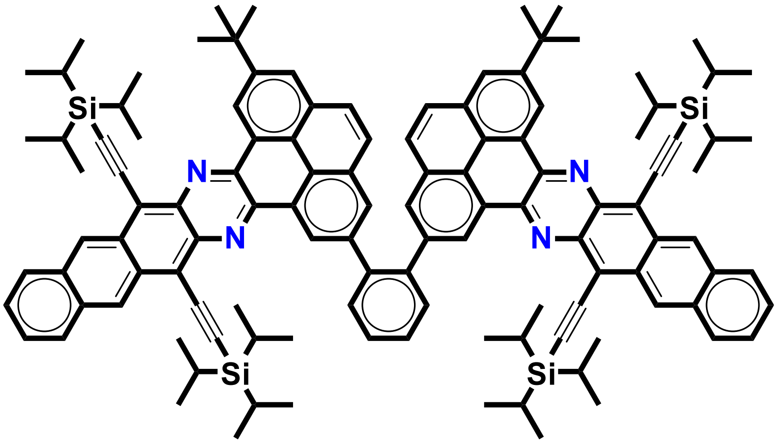

A solution of 1,2-diiodobenzene (11 mg, 0.033 mmol) and the compound **E** (75 mg, 0.079 mmol), $PdCl_2$(dppf) (4 mg, 0.005 mmol) as a catalyst, K_3PO_4 as the base (35 mg, 0.165 mmol) and THF/water (3 : 1 v/v) as the reaction medium. At the pulsed microwave power level of 100 W (80 °C, 120 PSI, 11 min). The product was extracted with CH_2Cl_2 (3 x 30 mL), dried over sodium sulfate and the organic phase was removed by rotary evaporation. The resulting solids were loaded onto a chromatographic column (eluent: mixture hexane : dichloromethane : diethyl ether 10:1:1). The resulting solids were precipitated until obtain the desired product as bright dark solid (14.5 mg, 25 %).

^{1}H NMR (400 MHz,CD_2Cl_2): 9.95 (d, *J* = 1.7 Hz, 2H), 9.72 (d, *J* = 1.9 Hz, 2H), 9.63 (s, 2H), 9.58 (s, 2H), 8.19 – 8.10 (m, 6H), 7.90 – 7.83 (m, 4H), 7.74 – 7.69 (m, 2H), 7.65 (d, *J* = 9.0 Hz, 2H), 7.62 – 7.57 (m, 4H), 7.47 (d, *J* = 9.0 Hz, 2H), 1.58 (s, 18H), 1.46 – 1.40 (m, 42H), 1.37 – 1.32 (m, 42H); ^{13}C NMR (101 MHz, CD_2Cl_2): 150.70, 146.04, 145.84, 143.01, 141.18, 140.75, 140.40, 140.08, 133.20, 133.11, 132.95, 132.54, 132.50, 131.79, 131.70, 131.03, 129.97, 129.59, 128.91, 128.41, 127.76, 127.28, 127.16, 126.73, 125.50, 124.91, 123.41, 122.93, 120.91, 120.58, 108.90, 108.73, 106.16, 104.19, 104.07, 35.68, 31.81, 19.33, 19.17, 12.20, 12.15.MS (MALDI): Calcd $C_{118}H_{131}N_4Si_4$: $[M+H]^+$:1715.9442 Found $[M+H]^+$:1715.9328. MS (MALDI): Calcd $C_{118}H_{130}AgN_4Si_4$: $[M+Ag]^+$:1821.8415 Found $[M+Ag]^+$:1821.8333 .

Synthesis of the compound *p*-TAD

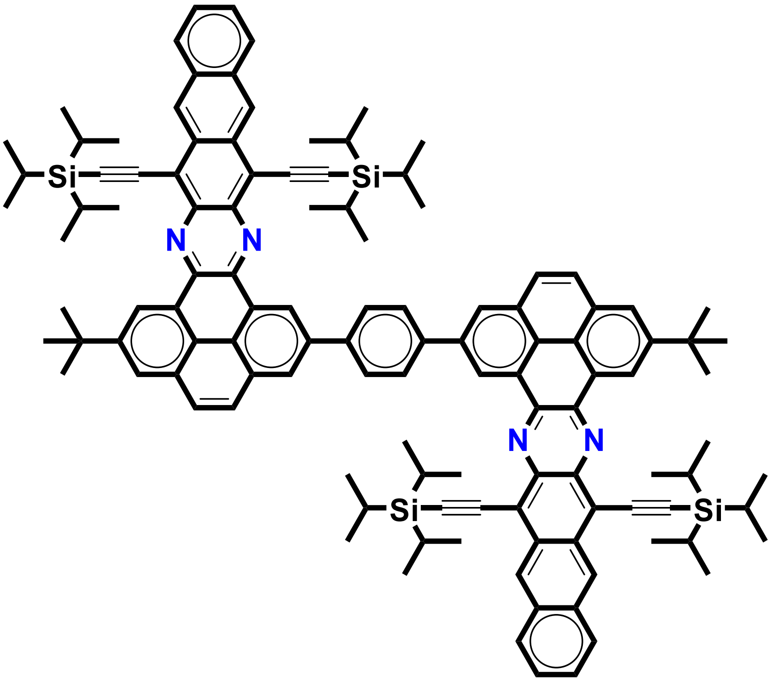

A solution of 1,4-diiodobenzene (10 mg, 0.033 mmol) and the compound **E** (75 mg, 0.079 mmol), $PdCl_2$(dppf) (4 mg, 0.005 mol) as a catalyst, K_3PO_4 as the base (32 mg, 0.151mmol) and THF/water (3 : 1 v/v) as the reaction medium. At the pulsed microwave power level of 100 W (80 °C, 120 PSI, 11 min). The product was extracted with CH_2Cl_2 (3 x 30 mL), dried over sodium sulfate and the organic phase was removed by rotary evaporation. The resulting solids were loaded onto a chromatographic column (eluent: mixture hexane : dichloromethane : diethyl ether 10:1:1). The resulting solids were precipitated until obtain the desired product as bright dark solid (12.2 mg, 23%).

^{1}H NMR (400 MHz,CD_2Cl_2): 9.98 (d, *J* = 1.9 Hz, 2H), 9.83 (d, *J* = 2.0 Hz, 2H), 9.59 (d, *J* = 22.1 Hz, 4H), 8.59 (d, *J* = 1.9 Hz, 2H), 8.38 (d, *J* = 2.0 Hz, 2H), 8.19 – 8.11 (m, 12H), 7.62 – 7.54 (m, 4H), 1.71 (s, 18H), 1.48 – 1.43 (m, 42H), 1.38 – 1.31 (m, 42H); ^{13}C NMR (101 MHz, $CDCl_3$): 150.67, 145.94, 145.78, 140.99, 140.28, 140.21, 140.06, 133.10, 132.97, 132.63, 132.13, 131.82, 130.45, 129.79, 129.28, 129.03, 128.94, 128.89, 128.27, 127.50, 127.23, 127.16, 126.94, 126.93, 126.24, 125.14, 124.46, 123.79, 120.95, 120.64, 108.68, 108.45, 104.25, 104.10, 100.29, 35.82, 32.09, 19.52, 19.41, 12.14, 12.07.MS (MALDI): Calcd $C_{118}H_{131}N_4Si_4$: $[M+H]^+$:1715.9442 Found $[M+H]^+$:1715.9371. MS (MALDI): Calcd $C_{118}H_{130}AgN_4Si_4$: $[M+Ag]^+$:1821.8415 Found $[M+Ag]^+$:1821.8312.

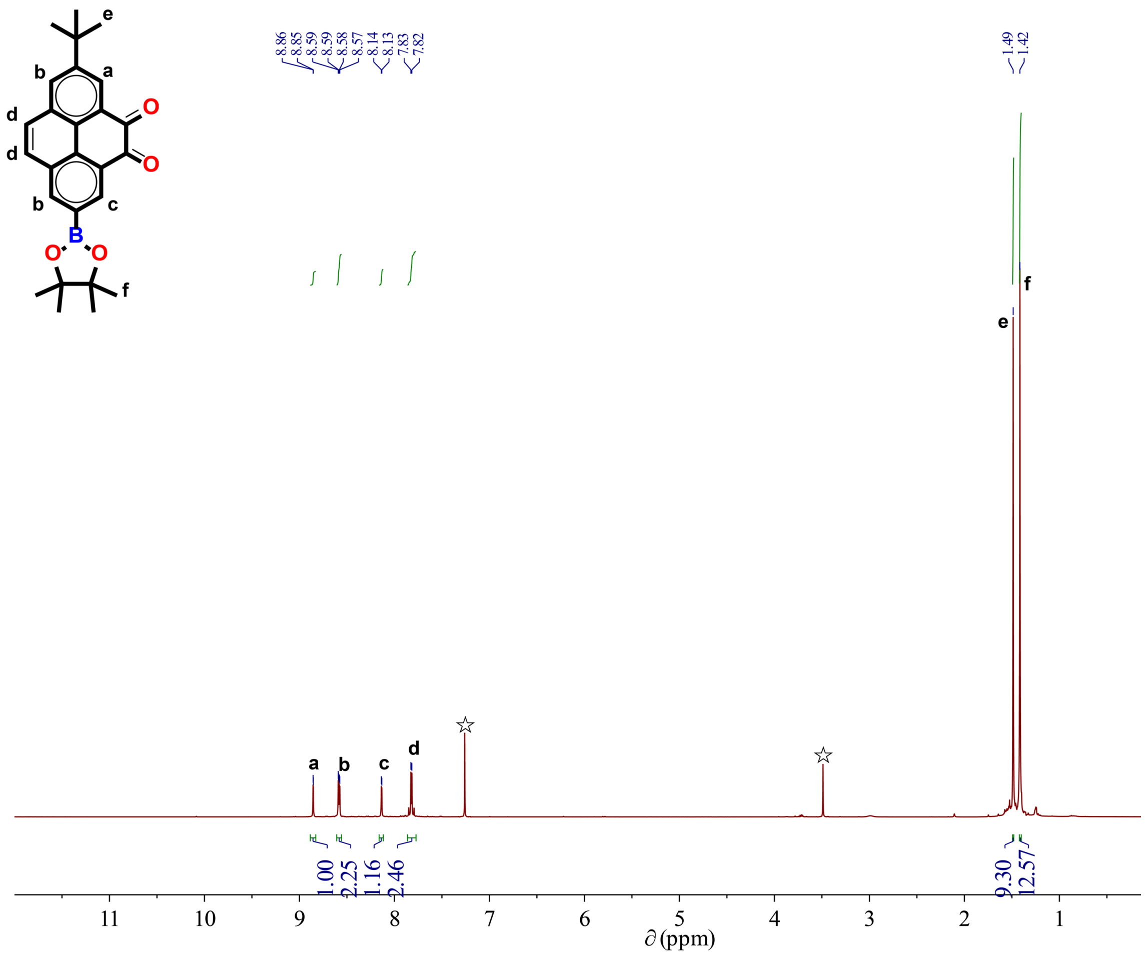

Figure S34. ^{1}H-NMR of the compound **A** in $CDCl_3$.

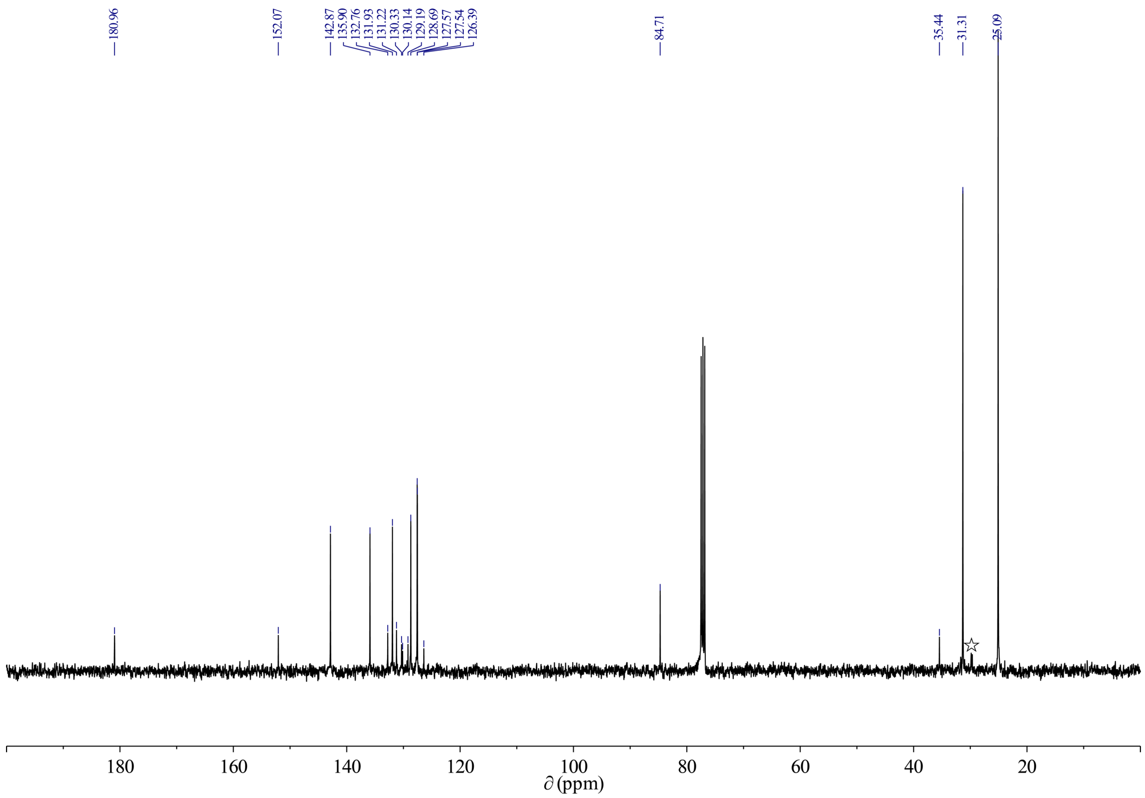

Figure S35. ^{13}C-NMR of the compound **A** in $CDCl_3$

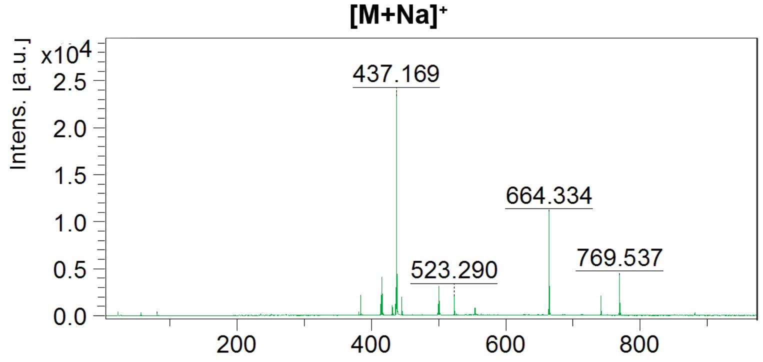

Figure S36. MALDI-TOF of the compound **A**.

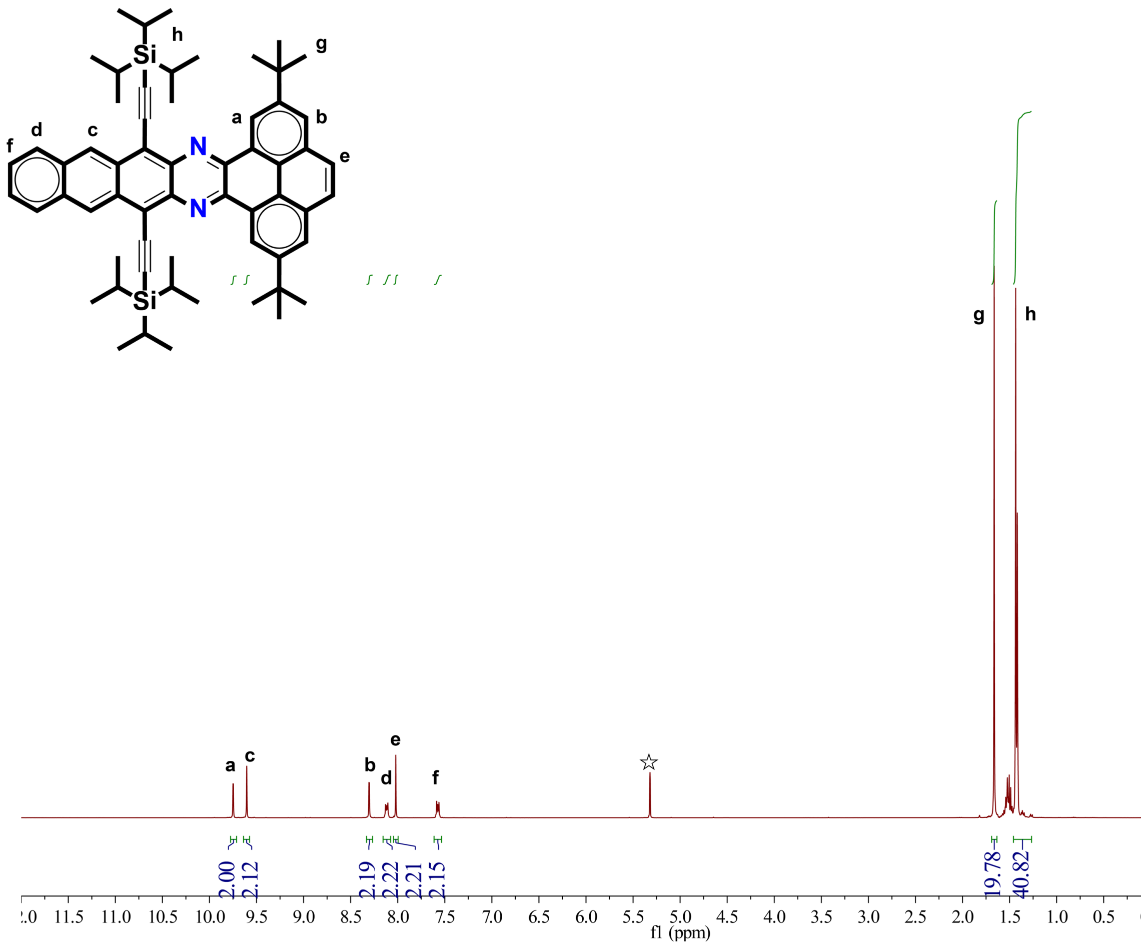

Figure S37. ^{1}H NMR of the compound **DAM** in CD_2Cl_2.

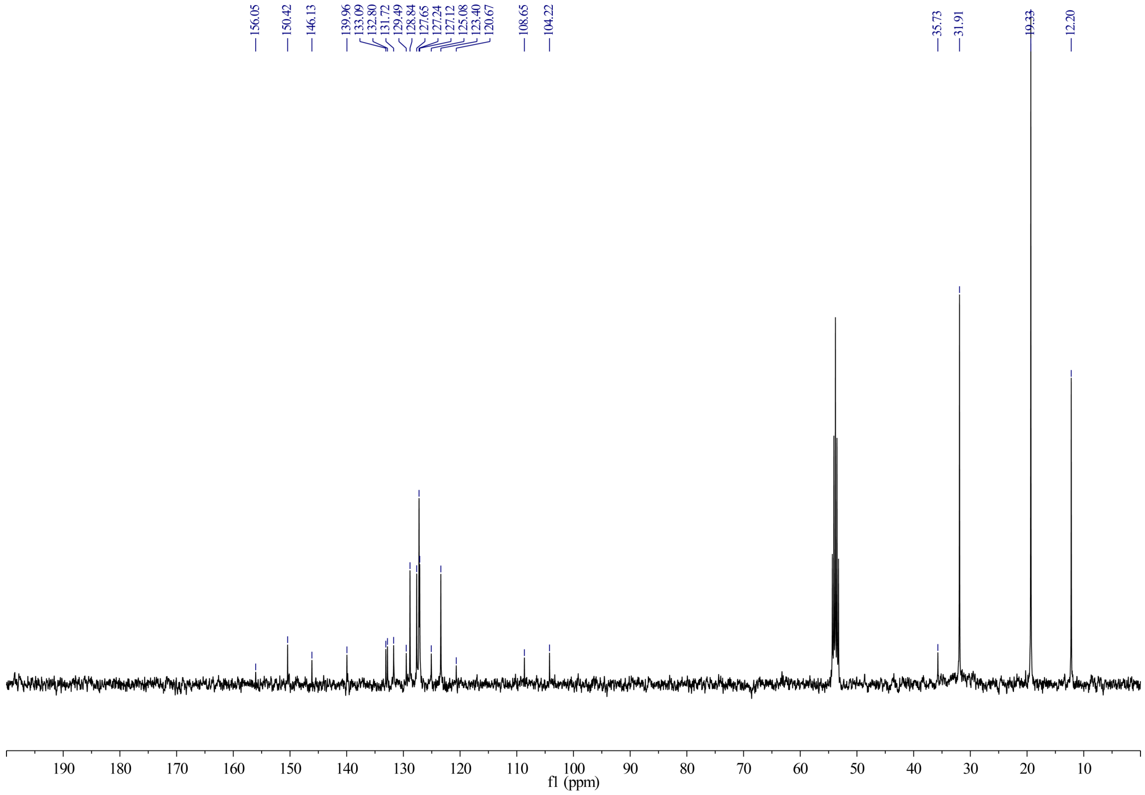

Figure S38. ^{13}C NMR of the compound **DAM** in CD_2Cl_2

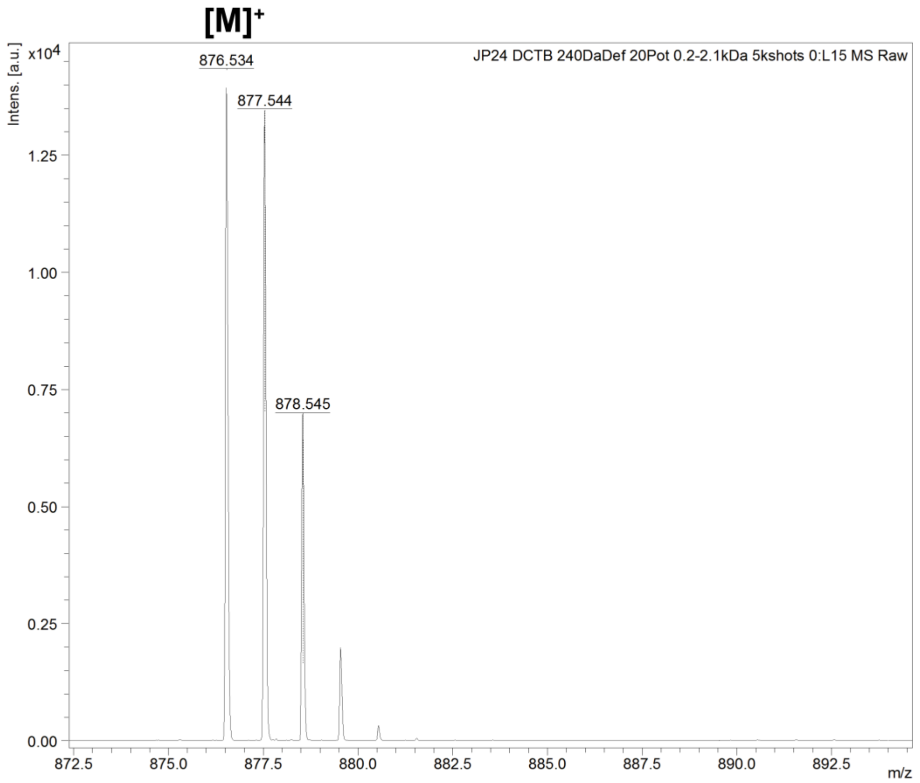

Figure S39. MALDI-TOF of the compound **DAM**.

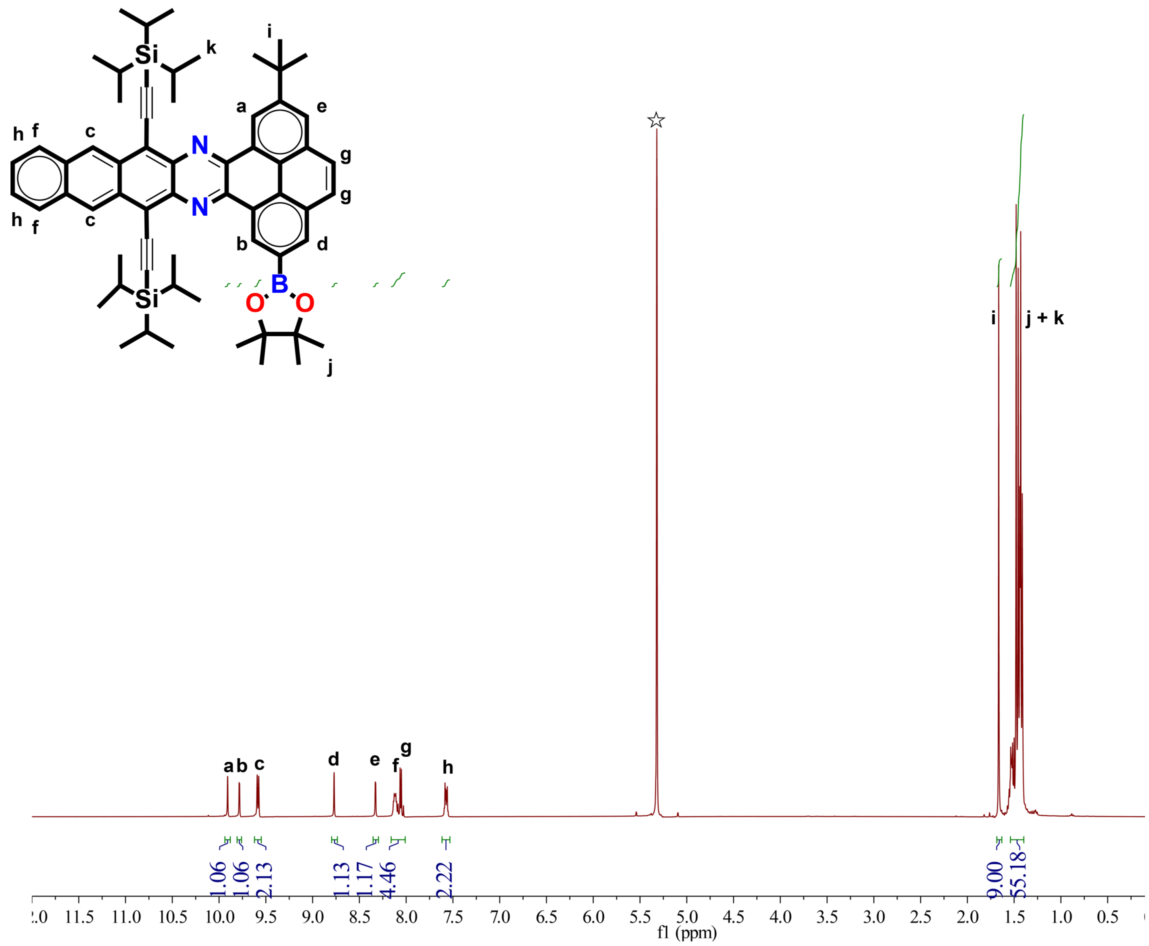

Figure S40. ^{1}H NMR of the compound **E** in CD_2Cl_2.

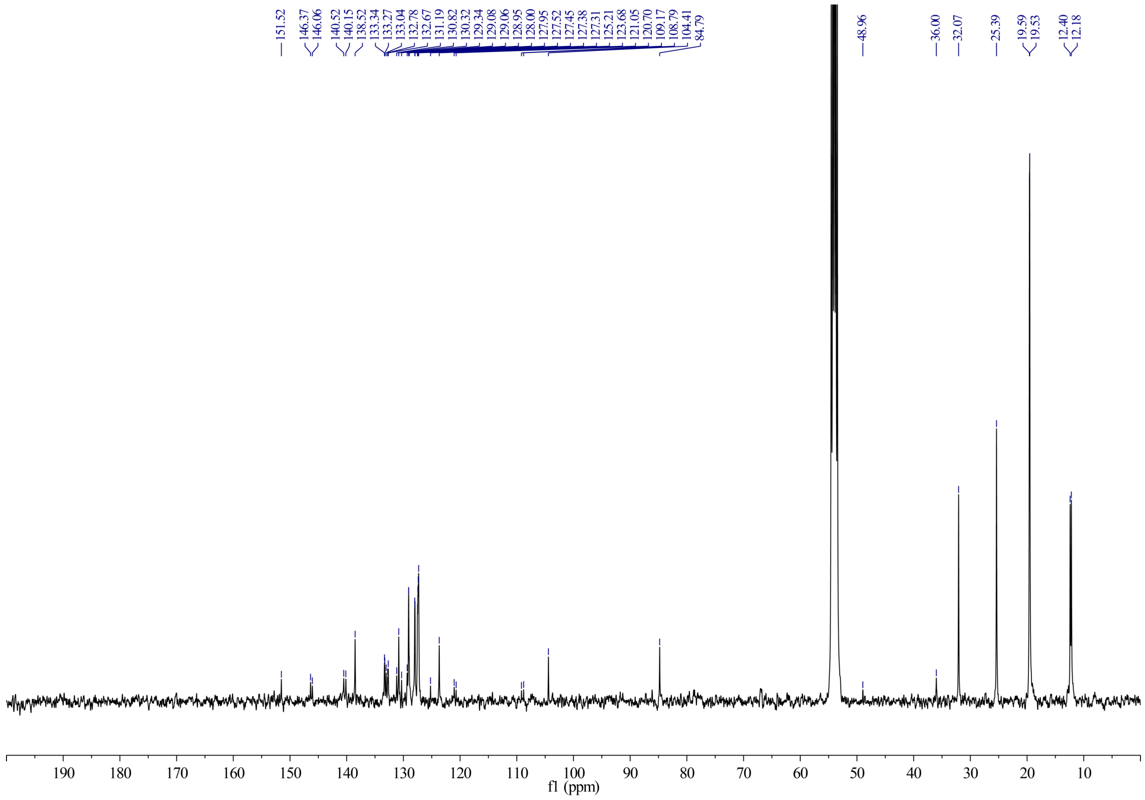

Figure S41. ^{13}C NMR of the compound **E** in CD_2Cl_2

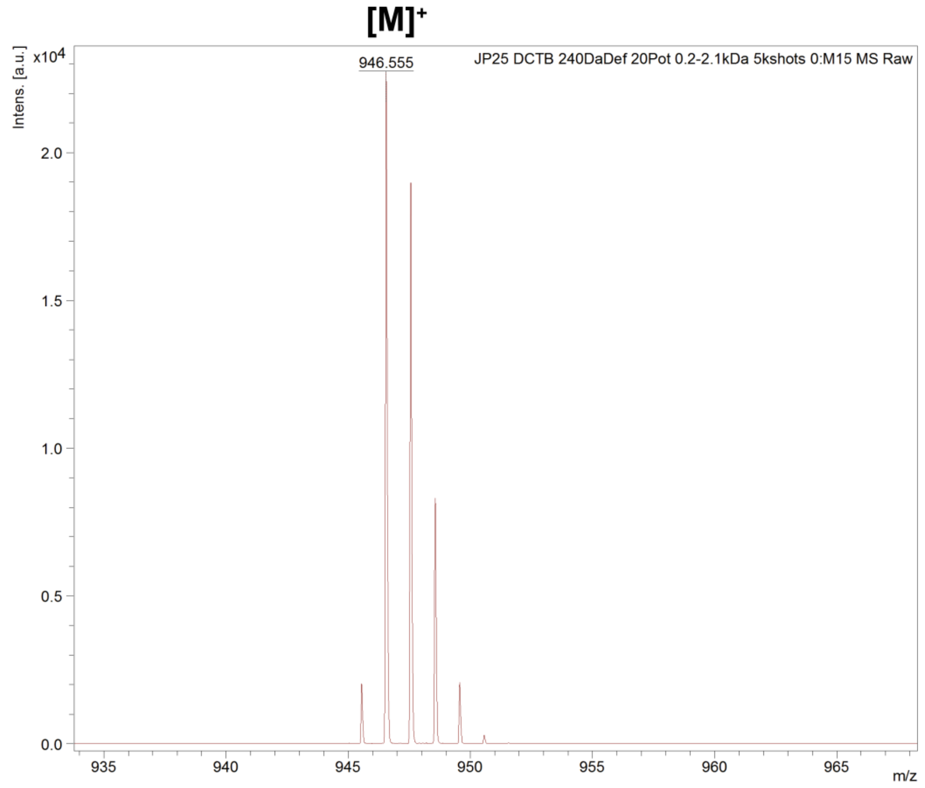

Figure S42. MALDI-TOF of the compound **E**

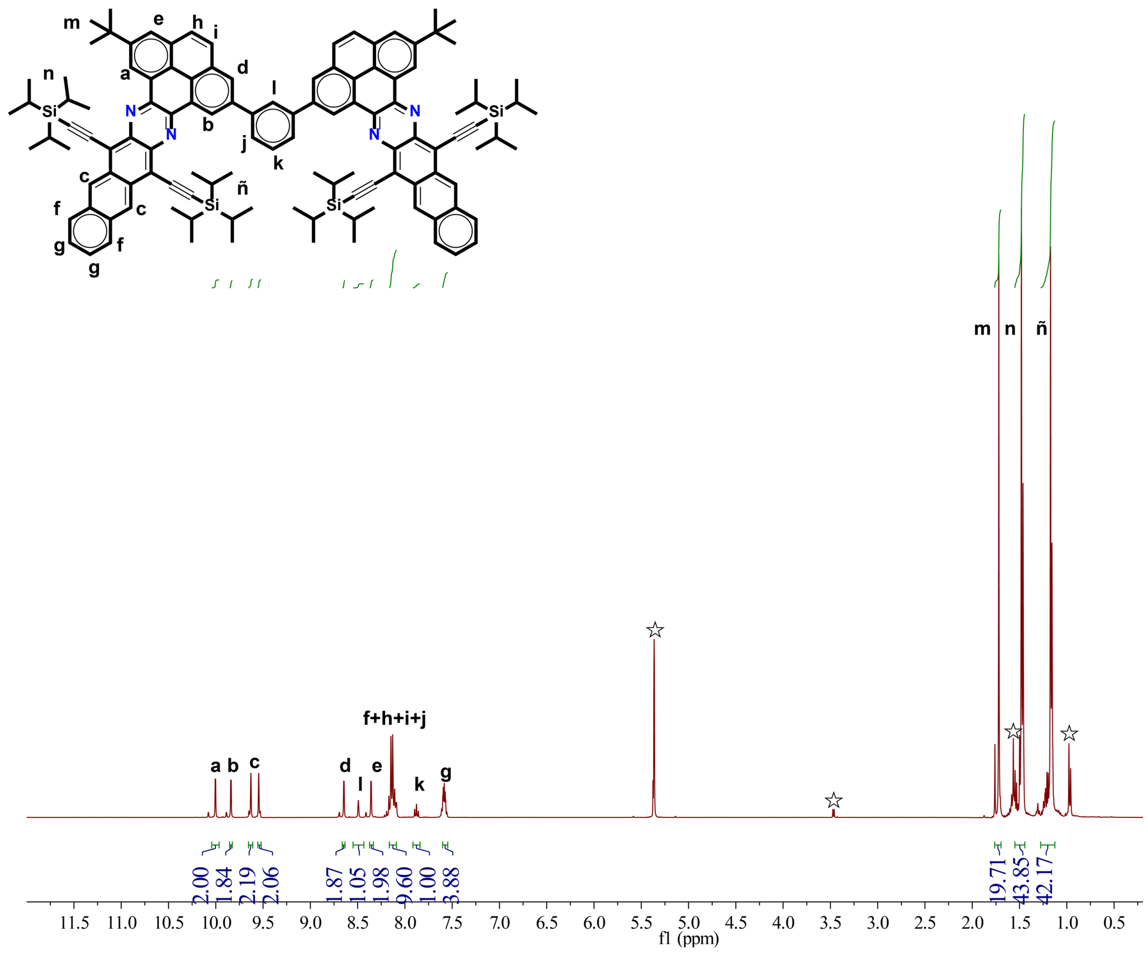

Figure S43. ^{1}H NMR of the compound ***m*-DAD** in CD_2Cl_2.

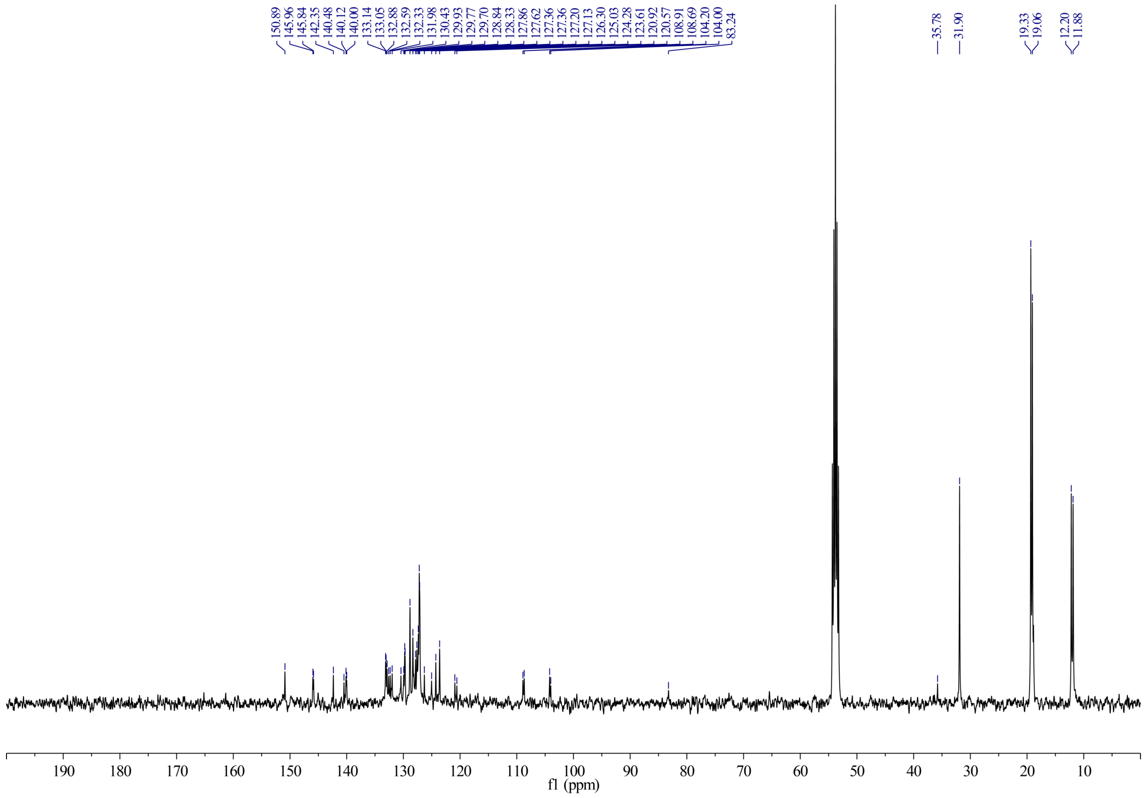

Figure S44. ^{13}C NMR of the compound ***m*-DAD** in CD_2Cl_2

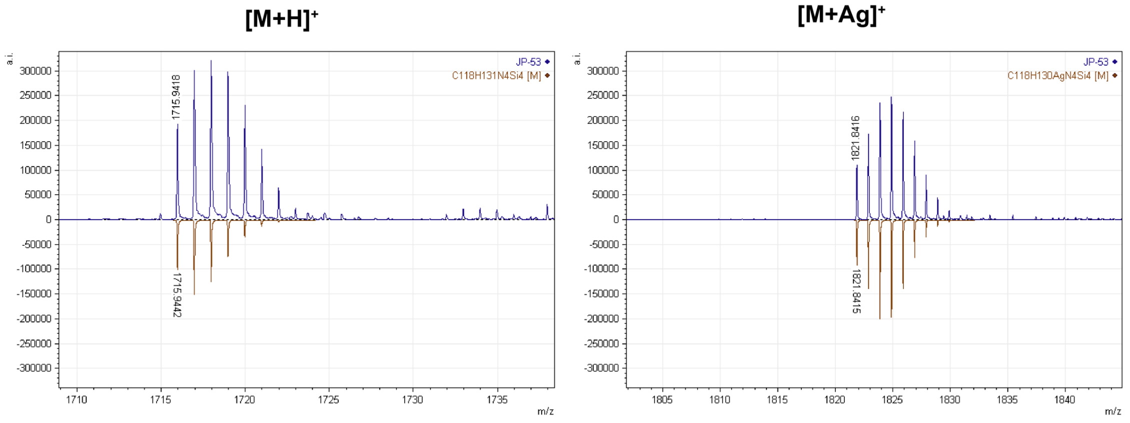

Figure S45. MALDI-TOF of the compound ***m*-DAD**.

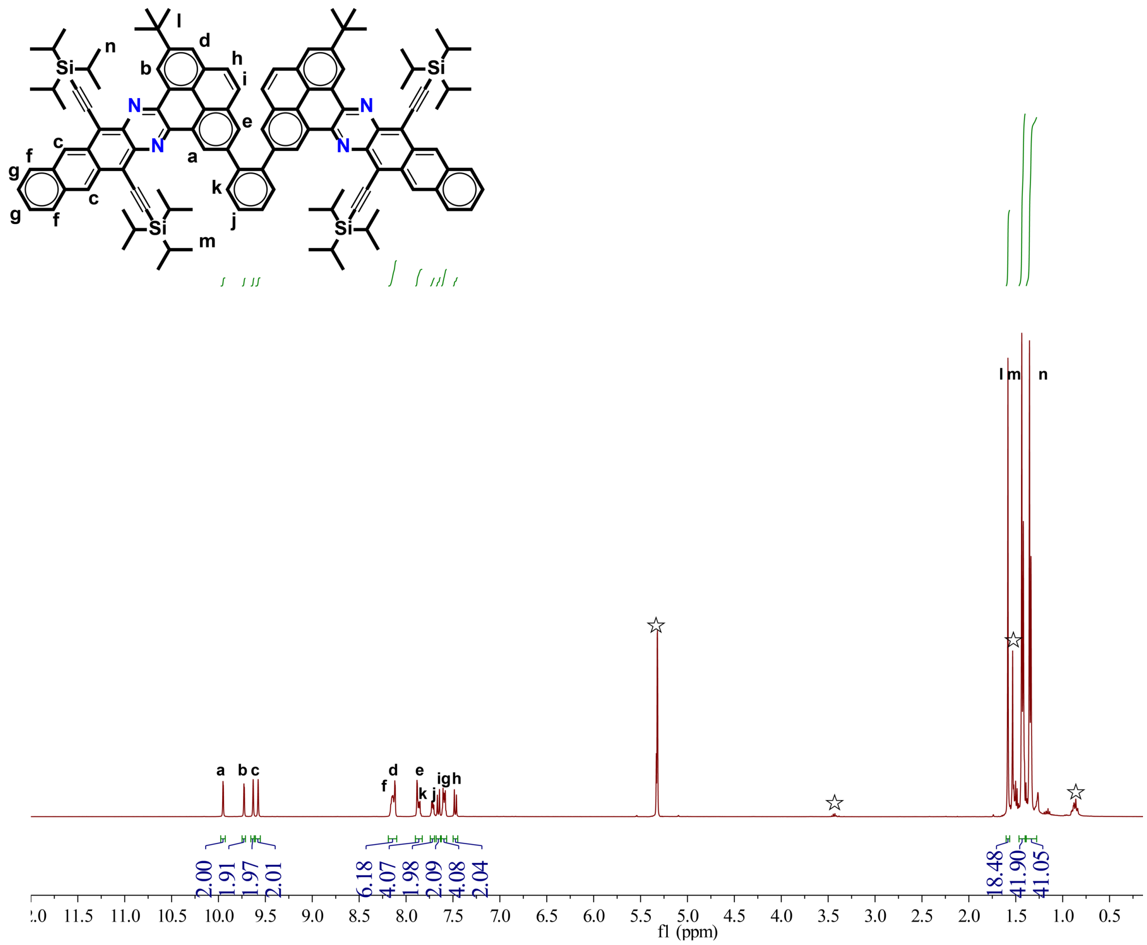

Figure S46. ^{1}H NMR of the compound ***o*-DAD** in CD_2Cl_2

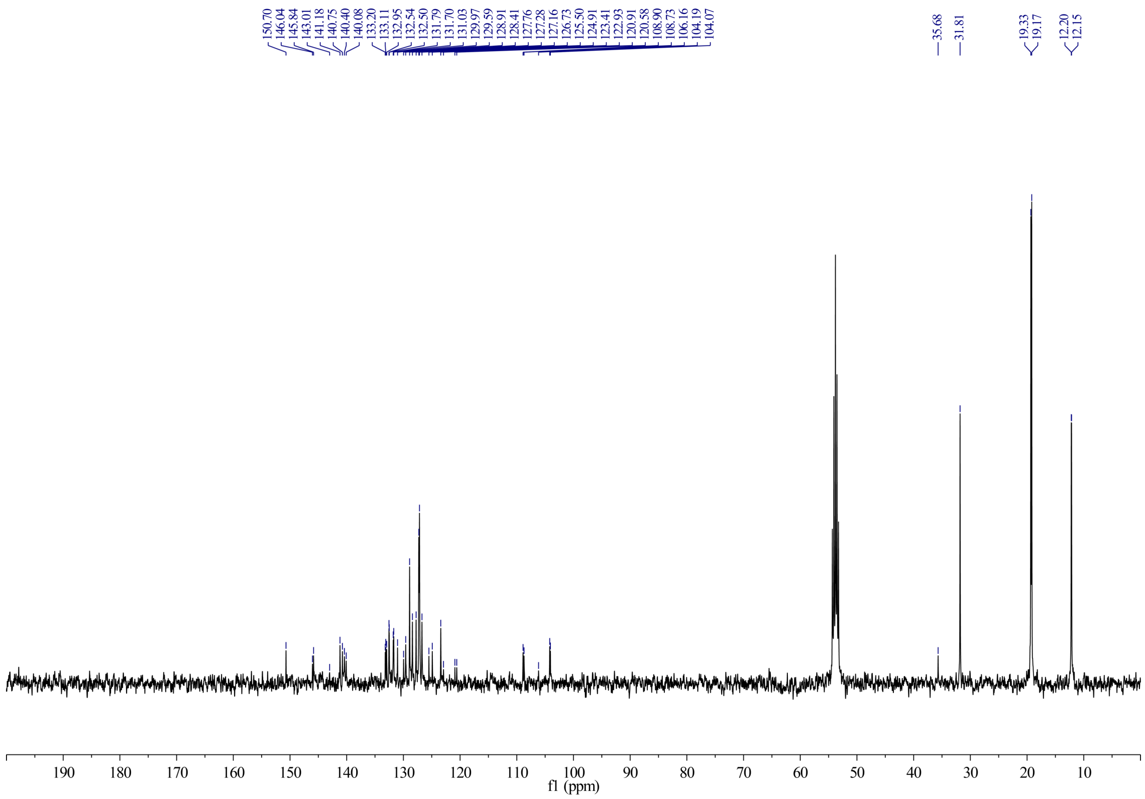

Figure S47. ^{13}C NMR of the compound ***o*-DAD** in CD_2Cl_2

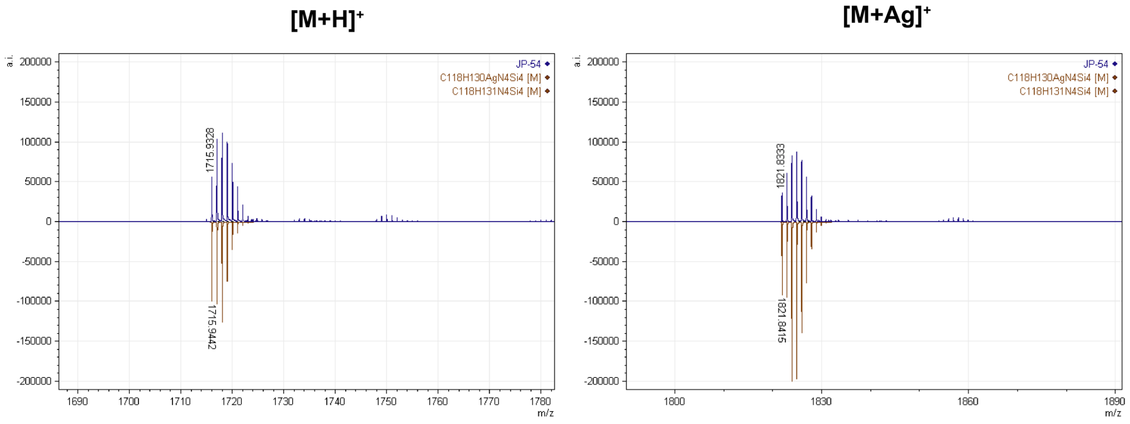

Figure S48. MALDI-TOF of the compound ***o*-DAD**.

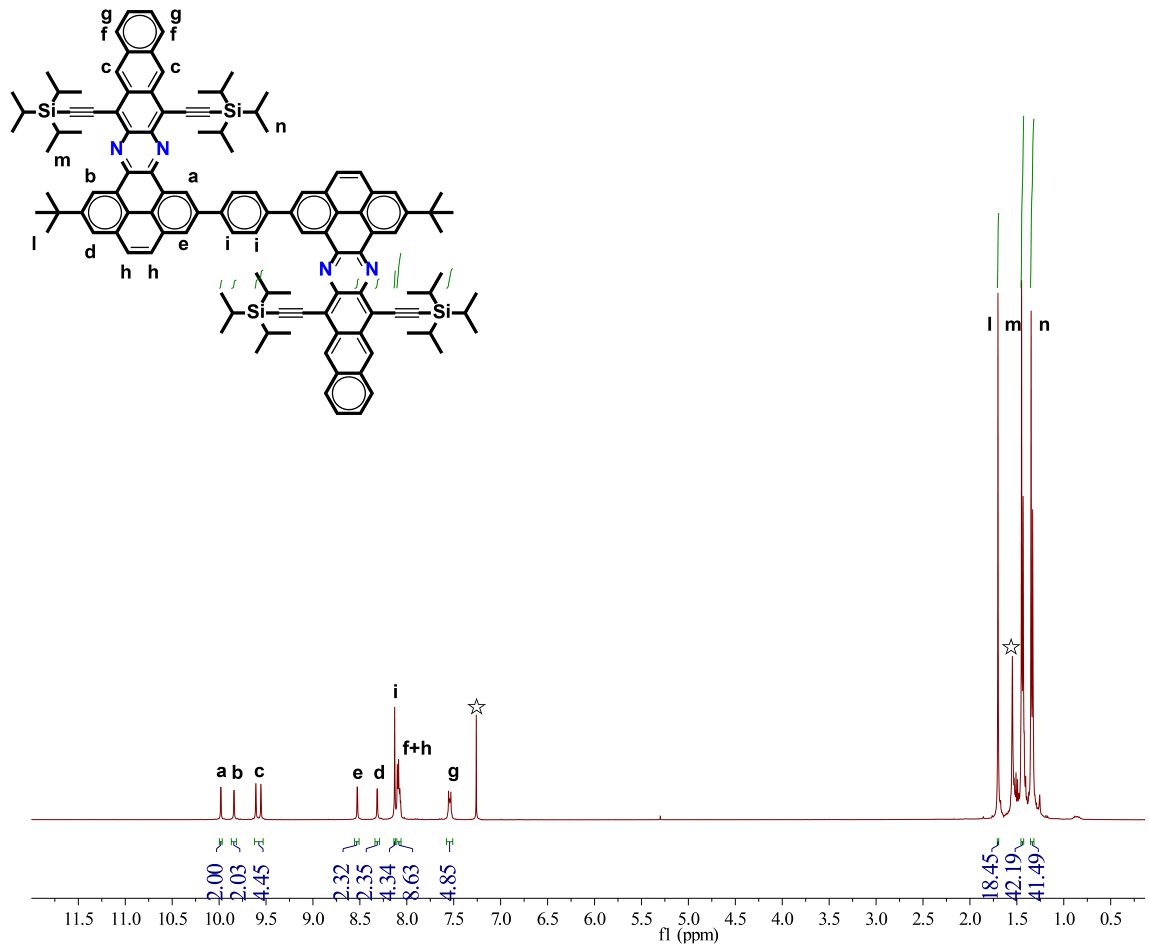

Figure 49. ^{1}H NMR of the compound ***p*-DAD** in $CDCl_3$.

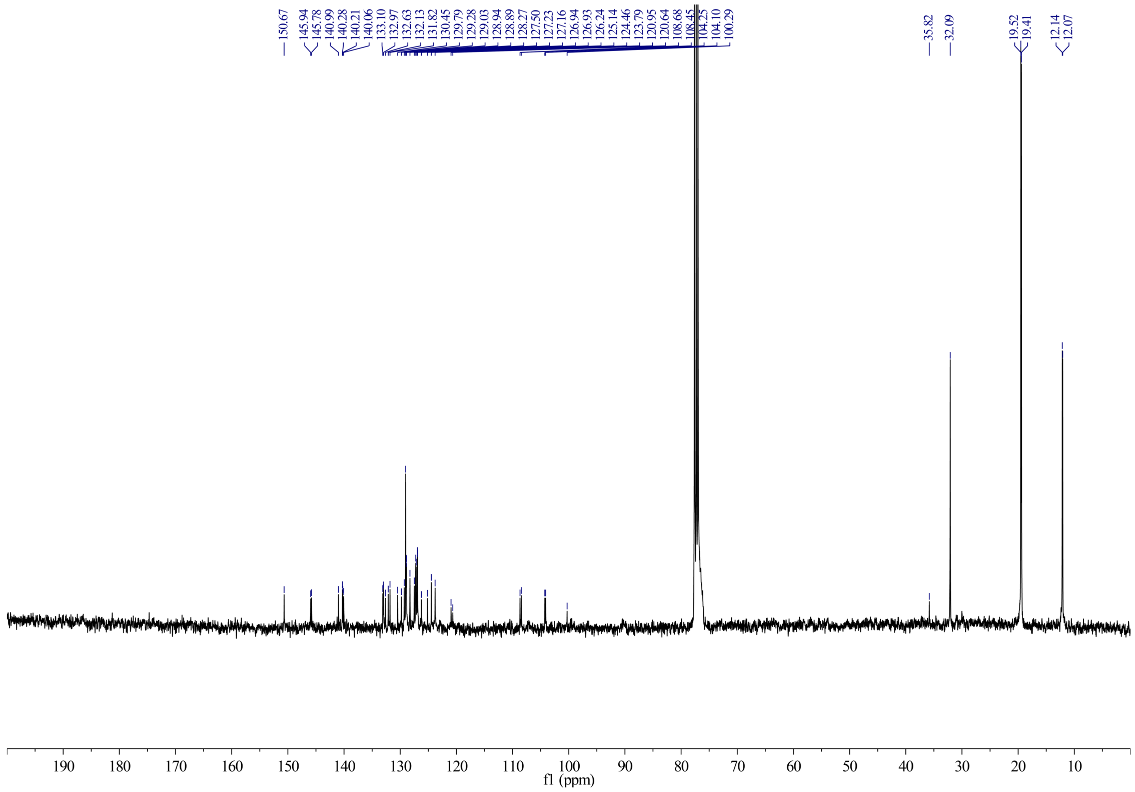

Figure S50. ^{13}C NMR of the compound ***p*-DAD** in $CDCl_3$.

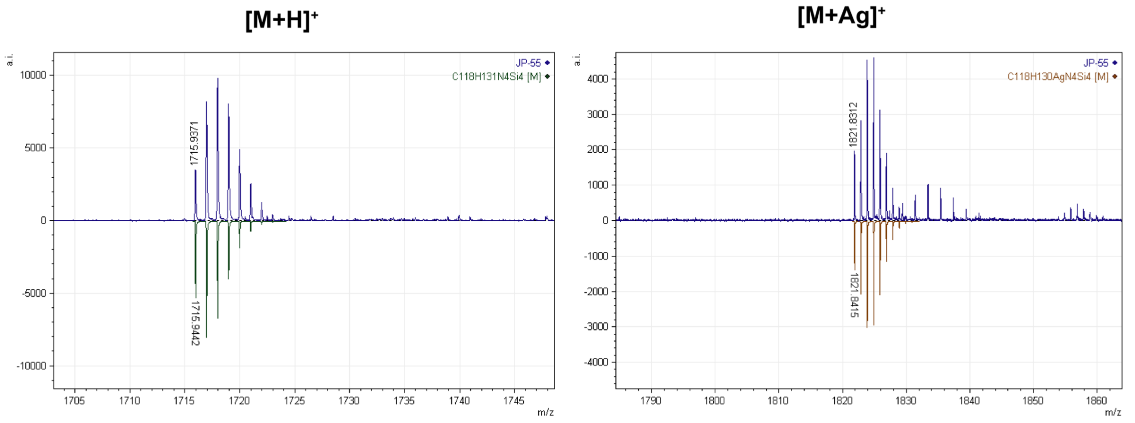

Figure S51. MALDI-TOF of the compound ***p*-DAD**.

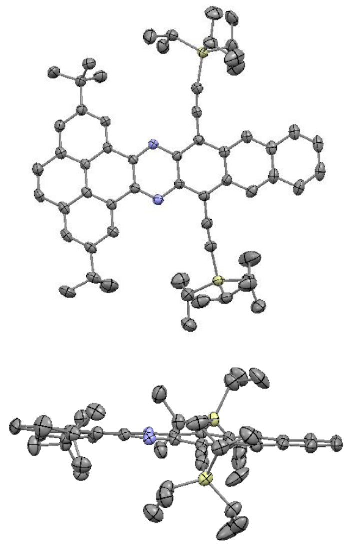

Figure S52. Two views for the X-ray crystal structure of **DAM**. Crystallographic data for DAM are deposited with the Cambridge Crystallographic Data Centre under reference number CCDC-1945880.

References

[1] (a) B. S. Basel, Zirzlmeier, J., Hetzer, C., Reddy, S. R., Phelan, B. T., Krzyaniak, M. D., Volland, M. K., Coto, P. B., Young, R. M., Clark, T., Thoss, M., Tykwinski, R. R., Wasielewski, M. R., Guldi, D. M., *Chem.* **2018**, *4*, 1092; (b) I. Papadopoulos, J. Zirzlmeier, C. Hetzer, Y. J. Bae, M. D. Krzyaniak, M. R. Wasielewski, T. Clark, R. R. Tykwinski, D. M. Guldi, *J. Am. Chem. Soc* **2019**, *141*, 6191-6203.

[2] (a) B. S. Basel, J. Zirzlmeier, C. Hetzer, B. T. Phelan, M. D. Krzyaniak, S. R. Reddy, P. B. Coto, N. E. Horwitz, R. M. Young, F. J. White, F. Hampel, T. Clark, M. Thoss, R. R. Tykwinski, M. R. Wasielewski, D. M. Guldi, *Nat. Commun.* **2017**, *8*, 15171; (b) S. Lukman, K. Chen, J. M. Hodgkiss, D. H. P. Turban, N. D. M. Hine, S. Dong, J. Wu, N. C. Greenham, A. J. Musser, *Nat. Commun.* **2016**, *7*, 13622; (c) S. Lukman, A. J. Musser, K. Chen, S. Athanasopoulos, C. K. Yong, Z. Zeng, Q. Ye, C. Chi, J. M. Hodgkiss, J. Wu, R. H. Friend, N. C. Greenham, *Adv. Funct. Mater.* **2015**, *25*, 5452-5461.

[3] It should be mentioned that the lifetime of $(S_1)_{SOL}$ is shorter in the more polar solvents than in the more apolar solvents.

[4] J. Zirzlmeier, Lehnherr, D., Coto, P. B., Chernick, E. T., Casillas, R., Basel, B. S., Thoss, M., Tykwinski, R. R., Guldi, D. M., *Proc. Natl. Acad. Sci.* **2015**, *112*, 5325-5330.

[5] S. Grimme, J. G. Brandenburg, C. Bannwarth, A. Hansen, *J. Chem. Phys.* **2015**, *143*, 054107.

[6] F. Neese, *Wiley Interdiscip. Rev. Comput. Mol. Sci.* **2012**, *2*, 73-78.

[7] A.-C. Bédard, A. Vlassova, A. C. Hernandez-Perez, A. Bessette, G. S. Hanan, M. A. Heuft, S. K. Collins, *Chem.: Eur. J* **2013**, *19*, 16295-16302.

[8] A. L. Appleton, S. Miao, S. M. Brombosz, N. J. Berger, S. Barlow, S. R. Marder, B. M. Lawrence, K. I. Hardcastle, U. H. F. Bunz, *Org. Lett.* **2009**, *11*, 5222-5225.
